# Scottish priorities for dementia and brain health research—results of a national research priority setting exercise

**DOI:** 10.1093/ageing/afag261

**Published:** 2026-10-20

**Authors:** Fenna Maria Alexandra Lunter, Winnie Henry, David Ross, Jacqui Kerr, Emma Law, Stephen Makin, Alison Mckean, Tom C Russ, Lucy Stirland, Susan Deborah Shenkin, Jennifer Kirsty Burton, Terence J Quinn

**Affiliations:** School of Cardiovascular and Metabolic Health, University of Glasgow, Glasgow, Scotland, UK; Public Contributor, NHS Research Scotland Neuroprogressive and Dementia Network, Edinburgh, UK; Public Contributor, NHS Research Scotland Neuroprogressive and Dementia Network, Edinburgh, UK; NHS Research Scotland Neuroprogressive and Dementia Network, Edinburgh, UK; NHS Research Scotland Neuroprogressive and Dementia Network, Edinburgh, UK; School of Medicine, Medical Sciences and Nutrition (Centre for Rural Health), University of Aberdeen, Aberdeen, Scotland, UK; Brain Health Scotland, Alzheimer Scotland, Edinburgh, UK; NHS Research Scotland Neuroprogressive and Dementia Network, Edinburgh, UK; Ageing and Health, Usher Institute, Edinburgh, UK; Division of Psychiatry, The University of Edinburgh Centre for Clinical Brain Sciences, Kennedy Tower Royal Edinburgh Hospital, Morningside Terrace, Edinburgh EH10 5HF, UK; Ageing and Health, The University of Edinburgh Usher Institute of Population Health Sciences and Informatics, 9 Little France Road, Edinburgh EH16 4UX, UK; Advanced Care Research Centre, The University of Edinburgh Usher Institute of Population Health Sciences and Informatics, Edinburgh, UK; School of Cardiovascular and Metabolic Health, University of Glasgow, Glasgow, Scotland, UK; School of Cardiovascular and Metabolic Health, University of Glasgow, Glasgow, Scotland, UK

**Keywords:** brain health, dementia, prioritisation, priority setting, patient and public involvement, Scotland, older people

## Abstract

**Background:**

Dementia is a global challenge, but benefits from local, context-specific evidence. With finite resource, and numerous potential research directions, formal priority setting can help develop equitable and relevant research agendas. We used this approach to establish dementia and brain health research priority questions for Scotland.

**Methods:**

A steering board of professionals and experts through lived experience guided the process, following established research prioritisation frameworks. Research questions were collected through surveys and refined to create an interim list. This was further rationalised through Delphi-style ranking involving cross-disciplinary, cross-sectoral interest-holders. After reaching consensus, each research question was assessed for potential impact.

**Results:**

Between April and July 2025, 706 participants submitted 2677 questions. These were consolidated into an interim list of 142 questions. Two ranking rounds (*n* = 242, *n* = 252) reduced the list to 20 questions (10 brain health, 10 dementia). Interest-holders included people living with cognitive impairment (*n* = 48, 6·8%), current or previous caregivers (*n* = 217, 30·7%), public contributors (*n* = 261, 37·0%), and professionals (*n* = 134, 19·0%). Risk reduction was a leading brain health priority, while early diagnosis was a top dementia priority. Research around brain resilience and improving dementia care pathways was selected as having greatest potential impact.

**Conclusion:**

We established Scotland-specific research priorities for brain health and dementia, which we advocate to researchers, funders and decision-makers. Our national research prioritisation exercise received engagement from diverse interest-holders, ensuring a range of voices will inform future research.

## Key Points

- Priority setting exercises can help shape the research agenda.
- We present the first national research priority setting exercise to consider brain health alongside dementia.
- Priorities identified by public and professionals include research into risk factors, resilience and novel treatments.

## Introduction

The importance of dementia is recognised at national and international levels and research can provide the foundation for evidence-based and impactful, policy and practice [1, 2]. While some aspects of dementia are cross-cutting and universal, solutions designed for the specific needs of local health systems, social care infrastructures and cultures are most likely to have traction. Dementia represents the clinical manifestation of pathological processes that begin earlier in the life course. Complementing traditional dementia research, there is increasing interest in maintaining cognition and preventing decline, often described as ‘brain health’ [3, 4]. Both dementia and brain health are Scottish Government key policy areas.

Scotland is a country within the UK, with a population of 5·4 million and a National Health Service (NHS), where healthcare is delivered free at point of contact [5]. Dementia is now the second leading cause of death in Scotland, with an estimated 90 000 affected individuals [6, 7]. Scotland’s political, clinical and academic interest in dementia and brain health is evidenced by a national brain health research strategy [8], government investment in research capacity building, and development of the Scottish Brain Health Service Model [9].

The enthusiasm around dementia and brain health research must be tempered by the reality of limited financial resources, finite research capacity and a seemingly overwhelming array of potentially important research questions. Robust, equitable and transparent methods to allocate research resources are needed [10]. Formally prioritising research allows involvement of diverse interest-holders in such research decisions, including the public and experts by lived experience [11, 12]. In an ecosystem where most research is directly, or indirectly, funded by the public, it seems appropriate to consult the Scottish public on their priorities for future research. While this exercise should include people whose lives are currently impacted by dementia, in theory, the whole Scottish population is at risk of cognitive issues, and anyone concerned about brain health should be given the opportunity to shape the research agenda in this space.

A research priority setting exercise (RPSE) is a methodology in which end-users and beneficiaries of research, the interest-holders [13], are engaged in topic solicitation, prioritisation and framing of research questions [11]. A recent systematic review describing published RPSEs in dementia revealed geographical differences in priority topics, emphasising the need for context-specific prioritisation. It also reported no RPSE specific to brain health [14]. There has been a previous, limited-scope RPSE for dementia conducted in Scotland [15] and a more comprehensive James Lind Alliance (JLA) priority setting for the UK [16]. Both these are now over a decade old, and given the rapid change in our understanding and management of dementia, and recognising that prioritisation should be cyclical [17–19], a new RPSE was warranted.

Our objectives were:

i) To elicit feedback from diverse interest-holders around important questions for research in dementia and brain health for Scotland.
ii) To develop a consensus list of the highest priority questions for future dementia and brain health research.
iii) To inform a research agenda for funders, policy makers and the wider research community, so that finite resources are allocated to topics that matter most to the people concerned.

## Methods

The exercise was conducted according to pre-specified phases, but with flexibility to iteratively adapt to interest-holder feedback: (i) setting up and context; (ii) identifying research priorities; (iii) ranking research priorities; (iv) dissemination of research priorities and follow-up (Figure 1). Ethical approval was obtained from the University of Glasgow MVLS Ethics Board. (project number: 200240255).

**Figure 1:**
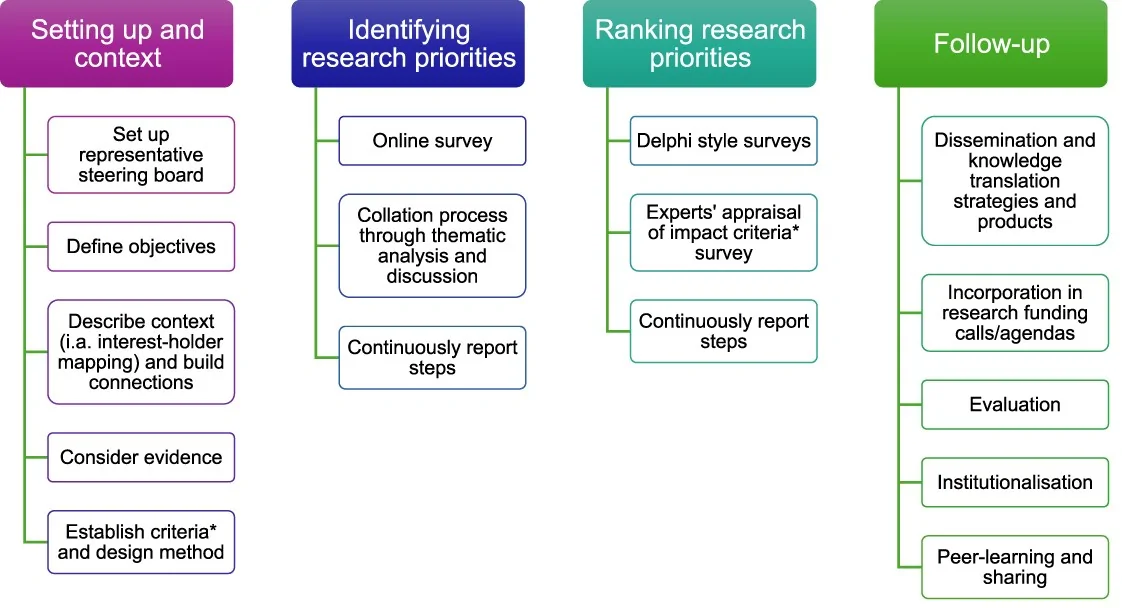
Elements per study phase based on frameworks by Tan *et al.* [17] and REPRISE [20]. The ‘Setting up and context’ phase is described in the methods. Objectives can be found in the introduction. All details of this phase are added as supplementary material. Specifics of the ‘Identifying research priorities’ phase and ‘Ranking research priorities’ can be found in the methods. Surveys were conducted online through Qualtrics. Information on the ‘follow-up’ phase can be found in the methods. ^*^Criteria adapted from World Health Organisation RPSE about dementia.

### Setting the context for priority setting

#### Framework

While there is no consensus on RPSE best practice, frameworks exist for various aspects and we used these to inform our approach to design, conduct and reporting. We selected the four-phase framework for national RPSEs [17] and reporting guideline for priority setting of health research (REPRISE) [20] as most relevant to our aims (Appendix S1). World Health Organisation (WHO) RPSE guidance was published after our study began, and we incorporated its principles where possible [10].

#### Scope

The study scope was proposed by the lead and refined with the steering board. Brain health over the life course and any cause of dementia were the co-primary focus. As there are various definitions of brain health, the steering board operationalised the term for the purposes of this exercise (Box 1).

Box 1 Definition of brain health as used for research prioritisation.

*Definition of brain health used for priority setting*

*The idea that, throughout the life course, cognitive damage can be prevented, and resilience can be built, by mitigating risk factors and optimising lifestyle choices, ultimately delaying or preventing neurodegenerative disease such as dementia.*

The scope included any form of research question. Interest-holder groups of interest were: professionals (health care professionals, researchers, third sector), people with lived experience of cognitive syndromes (personally or as a caregiver), and Scottish citizens with an interest in brain health.

#### Steering board

Using national networks covering dementia, brain health and older adult research, a steering board was formed to oversee all RPSE activity. We purposively recruited researchers, clinicians, third sector partners and people with lived experience. Efforts were made to ensure gender and geographic diversity (Appendix S2). Lived experience members were compensated through gift vouchers. The board met online at regular intervals with further correspondence by email and the core subgroup handling operational decisions.

### Identifying research priorities

Surveys were predominantly administered online via Qualtrics (browser version August 2025, Qualtrics LLC, USA) and designed for accessibility, using inclusiveness principles [21–23] and the NHS readability tool. Surveys were designed to take ten minutes or less, were anonymous and voluntary. Alternative formats were offered (e.g. phone or paper) for those not wishing, or able, to use an online tool. No reimbursement was given. Basic demographics of respondents and their free text feedback were collected. The process followed best practice in E-survey (Checklist for Reporting Results of Internet E-Surveys (CHERRIES [24]) (Appendix S3). All analyses were performed in Microsoft Excel, v2507, Microsoft, USA.

#### Survey A: collecting research questions

This questionnaire gave a brief introduction to brain health and research prioritisation, then allowed for submission of free text research questions, allowing up to ten suggestions per participant (full survey in Appendix S4). The survey was piloted with a lived experience group, then distributed through multiple channels, including: Join Dementia Research (JDR), professional, third sector, and charity mailing lists, social media and in person at relevant dementia meetings. Initial sampling was convenience-based, later supplemented with targeted outreach to people living with dementia and ethnic minority groups, as these populations appeared under-represented in interim data collection. Previously collected research questions during an exercise for a NIHR call were also included.

#### Interim priority list

Submitted questions were thematically categorised and refined through mixed inductive-deductive analysis by the core team. Duplicates, questions with an existing definitive evidence base (Appendix S5), topics that related to policy rather than research, and out-of-scope questions were identified by the core group and removed. Language was standardised. The draft interim list was reviewed and modified by the steering board before sharing with participants for final input.

### Ranking research priorities

To shorten the interim priority list, an online survey-based Delphi approach was chosen to allow equitable and speedy consensus building [10, 25, 26]. When consensus was reached, the resulting questions were appraised for impact criteria. Ranking analysis included stratification for interest-holder roles, gender and age groups.

#### Survey B and C: Delphi rounds

Interim list questions were categorised into eight themes and associated subthemes, with the order of presentation randomised. The questions were distributed using the same platforms as previously, including those who had contributed to the first survey. Participants could score each question with options of: ‘take OUT of list’; ‘Neutral’; or ‘Keep IN list’. These options were given values of 0, 0·5 and 1, respectively, to allow calculation of an average score per question. The steering board agreed to set the threshold for a new shortlist at 50 questions (+/− 5), without any preferential weighting.

For the second round Delphi process, participants selected and ranked their top ten priority research questions from the 50-item shortlist. An adjusted Borda count was used to generate a further revised list [27, 28]. The Borda count was calculated as an aggregated score where questions ranked 1 received a score of 10, ranked 2 received 9, etc. Questions that were not chosen were scored 0. The steering board agreed beforehand on a maximum of 20 items per list, with separate lists for brain health and dementia should the prioritised questions allow (Table 1).

**Table 1:** Top 10 priority research questions in brain health and dementia.

***Top 10 research priority questions: brain health*** *How much can people, especially those with high genetic or other risks for dementia, reduce their risk?* *What brain stimulating, and lifelong learning activities, are beneficial for brain health? (including amount, types, variety and suitability for different ages/abilities)* *How can we financially prepare our health care system for the future, considering the ageing population?* *Of the many factors contributing to brain health, which is the most important one?* *What is the role of diet in brain health, and how can we intervene (considering different stages of the life course, and specific types of food/drink—like ultra processed food)* *What is the role of sleep in brain health and how can we intervene?* *What is the role of physical activity in brain health and how can we intervene? (including amount and types of exercise)* *What is the role of stress and anxiety in brain health and how can we intervene? (including different sources, amounts and length of exposure)* *What are population-wide interventions to benefit brain health that can be implemented during childhood, mid-life and older age?* *Why do some people, especially those with high genetic or other risk on paper, keep good brain health in reality? (including brain resilience)* ***Top ten research priority questions: dementia*** *What are ways to diagnose dementia earlier than in current practice? (including identifying key early symptoms, and biomarkers)* *What new treatments for preventing or stopping dementia can be developed? (including acceptable side effects, optimal timing, applicability for subtypes of dementia)* *How can we optimise the brain health and dementia care pathways in Scotland? (including personalisation, follow-up, designing new ‘memory clinics’ and cross-disciplinary links)* *Are there existing drugs that can be repurposed to help prevent dementia?* *What support can be given to let people live independently and/or at home as long as possible?* *How can we improve care for people living with advanced dementia to maintain quality of life?* *What support do caregivers need and do current services meet their (differing) needs?* *How can we ensure that all health and social care professionals, not just dementia specialists, provide dementia friendly care?* *How can brain scans best be used in care? (including predicting dementia risk and following brain changes over time)* *What can we learn from other countries’ dementia and brain health care systems? (including assisted dying law)*

#### Survey D: expert appraisal of impact

Assessment of the potential impact of the chosen questions was performed by cross-disciplinary, cross-sectoral experts in the Scottish dementia research field, using WHO RPSE criteria [29] and considering five domains of: feasibility, burden reduction, novelty, implementation and equity (Appendix S6).

This assessment added depth to the priorities, recognising that not all questions, even in a list of ten, may be selected by research funders and commissioners. To allow for analysis and data visualisation, we followed an approach used in other RPSE where impact was scored using a five-point Likert scale, and agreement presented using a three-point scale.

### Dissemination of top ten research priorities and follow-up

#### Feedback

At all survey stages we invited feedback from respondents. This allowed us to address any technical difficulties, and to hear reflections of participants, including explanation of rationale for choices or suggested modifications to language.

#### Distribution channels

In collaboration with strategic partners, we developed a dissemination and implementation strategy, which includes a plain language summary for funders, third sector and public; social media content; and a webinar (Appendix S1) A new cycle, preceded by a review of current priorities, is anticipated in five to ten years, pending funding confirmation.

## Results

### Demographics of survey participants

The first three phases took place between February and August 2025. The follow-up and sustainability phases are ongoing. Survey A opened on 29 April, closed 26 May 2025, and was completed by 706 interest-holders, the majority participating through the JDR web platform (*n* = 564). In total, *n* = 48 (6·8%) self-identified as living with a cognitive syndrome (mild cognitive impairment or dementia) and *n* = 217 (30·7%) self-identified as a current or former caregiver. Out of 242 interest-holders participating in Survey B, 93·0% (*n* = 225) had participated in Survey A (*n* = 243, 96·4% for Survey C). There were more female participants than male participants, and most participants were aged between 50 and 69 years (Table 2).

**Table 2:** Demographics of survey respondents.

		Survey A collecting questions	Survey B Delphi round 1	Survey C Delphi round 2
Question	Answer options	*n* = 706	*n* = 242	*n* = 252
Interest-holder role	Person living with dementia	12 (1·7%)	3 (1·2%)	3 (1·2%)
	Person living with mild cognitive impairment	36 (5·1%)	14 (5·8%)	11 (4·4%)
	Caregiver	74 (10·5%)	20 (8·3%)	18 (7·1%)
	Former caregiver	143 (20·3%)	42 (17·4%)	52 (20·6%)
	Researcher	50 (7·1%)	12 (5·0%)	18 (7·1%)
	Clinician^a^	50 (7·1%)	12 (5·0%)	16 (6·4%)
	Third sector^b^	34 (4·8%)	8 (3·3%)	9 (3·6%)
	Scottish citizen with interest in brain health	261 (37·0%)	114 (47·1%)	104 (41·3%)
	Other^c^	46 (6·5%)	17 (7·0%)	21 (8·3%)
Type of living location	Large city	174 (24·6%)	52 (21·5%)	57 (22·6%)
	Suburb near a large city	133 (18·8%)	56 (23·1%)	51 (20·2%)
	Small city or town	254 (36·0%)	71 (29·3%)	86 (34·1%)
	Rural area	136 (19·3%)	62 (25·6%)	56 (22·2%)
	Other	3 (0·4%)	1 (0·4%)	1 (0·4%)
	Prefer not to say	6 (0·8%)	0 (−)	1 (0·4%)
Gender	Male	191 (27·1%)	75 (31·0%)	82 (32·5%)
	Female	509 (72·1%)	167 (69·0%)	170 (67·5%)
	Non-binary/third gender	0 (−)	0 (−)	0 (−)
	Prefer not to say	6 (0·8%)	0 (−)	0 (−)
Ethnicity	White	663 (93·9%)	233 (96·3%)	237 (94·0%)
	Mixed or multiple	5 (0·7%)	2 (0·8%)	2 (0·8%)
	Asian, Asian Scottish or Asian British	22 (3·1%)	5 (2·1%)	6 (2·4%)
	Caribbean or Black	4 (0·6%)	1 (0·4%)	1 (0·4%)
	Other	3 (0·4%)	0 (−)	3 (1·2%)
	Prefer not to say	9 (1·3%)	1 (0·4%)	3 (1·2%)
Age group	18–29 years old	32 (4·5%)	9 (3·7%)	16 (6·4%)
	30–39 years old	69 (9·8%)	15 (6·2%)	19 (7·5%)
	40–49 years old	76 (10·8%)	18 (7·4%)	17 (6·8%)
	50–59 years old	145 (20·5%)	43 (17·8%)	49 (19·4%)
	60–69 years old	214 (30·3%)	87 (36·0%)	80 (31·8%)
	70–79 years old	134 (19·0%)	56 (23·1%)	52 (20·6%)
	80–89 years old	29 (4·1%)	14 (5·8%)	18 (7·1%)
	90–99 years old	0 (−)	0 (−)	0 (−)
	>100 years old	0 (−)	0 (−)	0 (−)
	Prefer not to say	7 (1·0%)	0 (−)	1 (0·4%)
Participated previously	Yes^d^	n/a	225 (93·0%)	243 (96·4%)
	No	n/a	17 (7·0%)	9 (3·6%)

All categories are self-identified;

^a^Doctor, nurse, paramedic etc;

^b^Policy, charity, etc;

^c^Mostly (late) family member with dementia but not main caregiver, also former clinician, person with memory concerns, combination, social worker, industry;

^d^This category was merged from answers ‘Yes’ and ‘Yes, but I would like to see the explanation again’

### Research priorities

Survey A yielded 2575 submitted research questions, with a further 102 questions received from piloting and the steering board. A draft interim list of 139 questions was developed, with an additional 28 submissions added following the first surveys (Figure 2).

**Figure 2:**
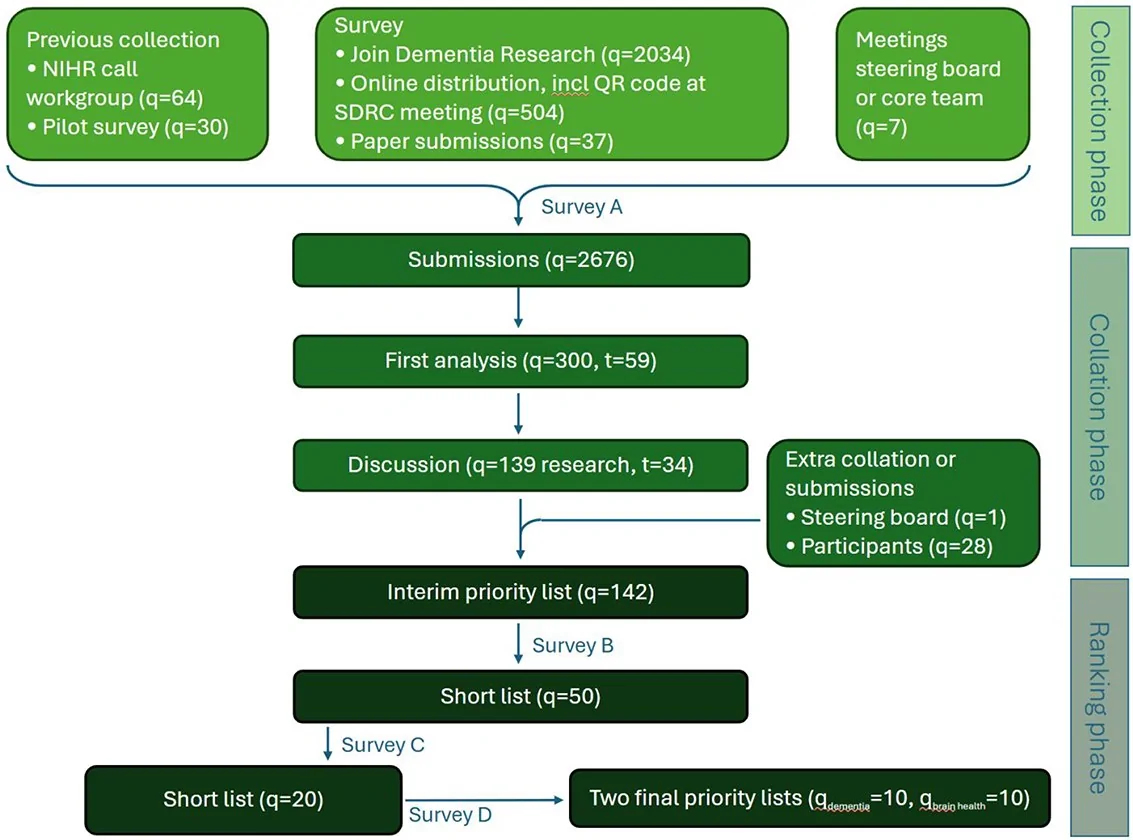
Flow chart of research priorities per study phase. q = questions, t = subthemes, NIHR, National Institute for Health and Care Research, SDRC, Scottish dementia research consortium.

The thematic analysis of submissions yielded 59 subthemes, reduced to 34 after discussion with the core team. The theme of ‘Pathology’ contained the most subthemes (*n* = 7) and questions (*n* = 36), while ‘Equity’ had the least subthemes (*n* = 2) and questions (*n* = 6) (Appendix S8).

Scores for survey B, the first Delphi round, ranged from 0·15 to 0·88, with a median of 0·55. The pre-defined cut-off of the highest-ranked 50 questions coincided with a natural threshold in distribution of scores, at a score of 0·6. Stratification for interest-holder groups (people with a cognitive syndrome, caregivers, professionals and the Scottish lay population) and gender showed no major differences in voting. Stratification by age group (18–39, 40–59, 60–79 and >80 years old) suggested differential voting by age. For the youngest group (*n* = 24), four of their top ten questions did not appear in the overall top fifty and were therefore not taken into the next Delphi round (Appendix S9).

Survey C produced adjusted Borda counts ranging from 57 to 1462 with a median of 182·5. ‘Natural cut-offs’ in the ranking were observed after positions 11, 14, 18 and 20. Again, stratification by interest-holder groups did not show any substantial differences in rankings. On review by the steering board, it was noted that the top 20 research questions contained an equal mix of topics that could be categorised as most pertinent to dementia, and most pertinent to brain health. So, a decision was made to offer two top ten lists, one for each.

### Expert appraisal of impact

The experts chosen for final impact appraisal (*n* = 17) represented various Scottish universities, disciplines and gender diversity (Appendix S7). Priority questions differed in their perceived impact (Figure 3A and 3B). For brain health, mean impact scores ranged from 0·51 to 0·69, and for dementia from 0·61 to 0·77. All mean impact scores were over 0·5, indicating general agreement that the questions were impactful. Dementia-related priorities generally received higher mean impact scores than brain health priorities. Among brain health priorities, ‘financially prepare for the future’ and ‘brain resilience’ tied for highest impact (mean impact score (i) = 0·69). For dementia, ‘optimising the care path’ ranked highest (i = 0·77), followed closely by both ‘care for advanced dementia’ and ‘learning from other countries’ (i = 0·74).

**Figure 3:**
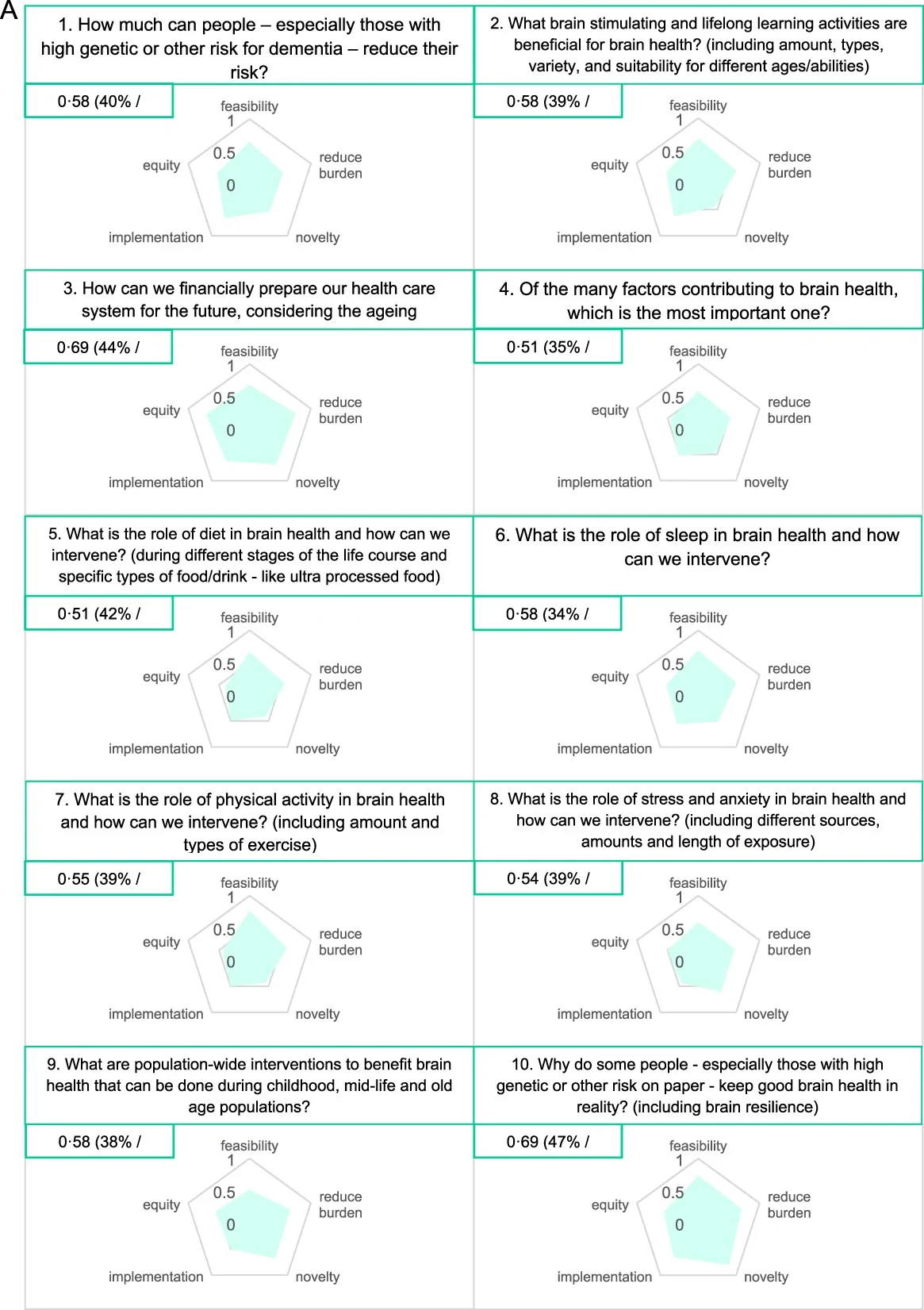

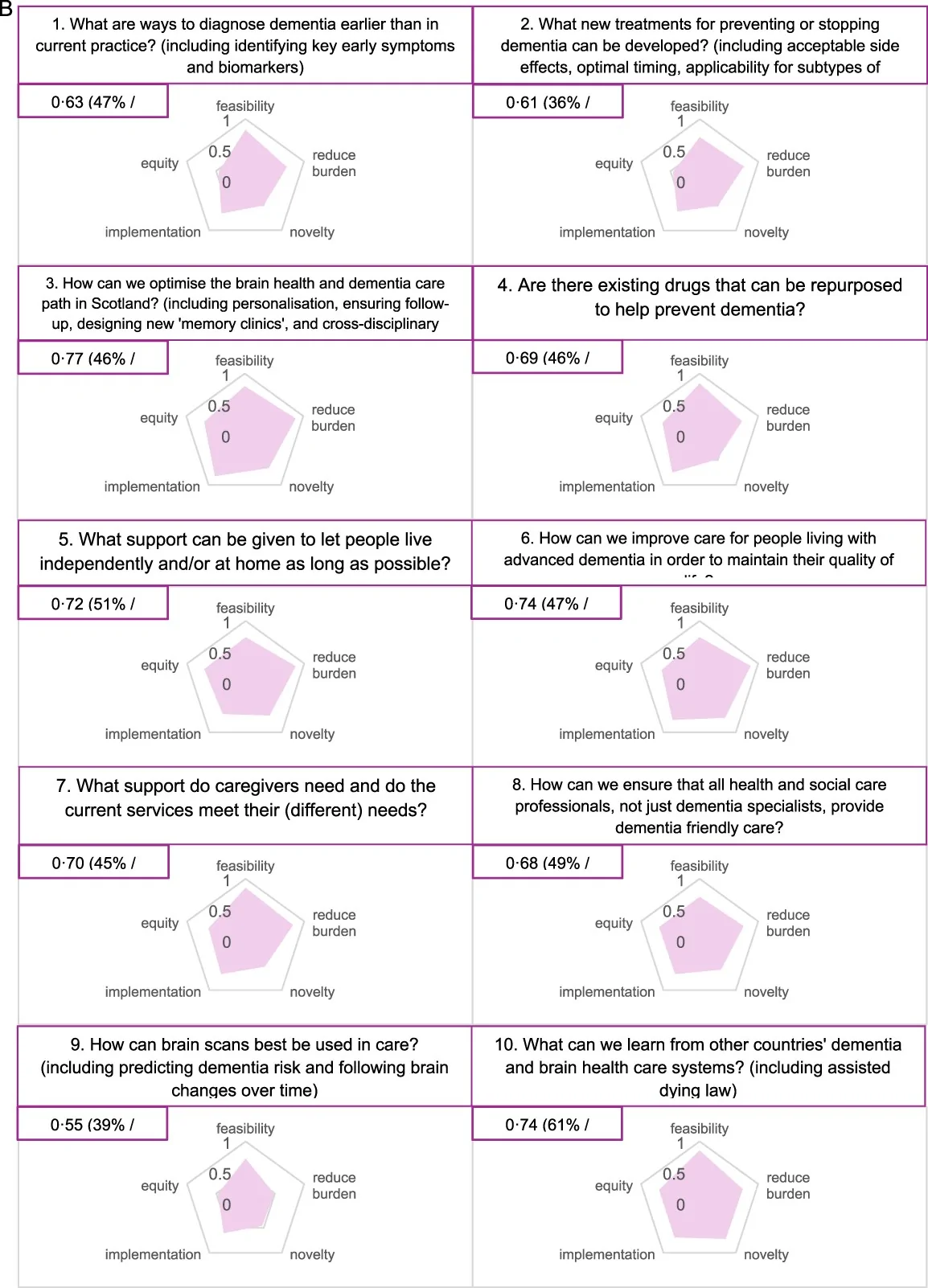
A. Final top 10 research priority list brain health. Box in top left shows overall impact score (five-point mean expert agreement/three-point mean expert agreement). The pentagon points are priority scores per impact criterion (Shah *et al.* [29]) impact was assessed across these five domains. B. Final top 10 research priority list dementia box in top left shows overall impact score (five-point mean expert agreement/three-point mean expert agreement). The pentagon points are priority scores per impact criterion (Shah *et al.* [29]). Impact was assessed across these five domains.

### Feedback

Free-text feedback was received from 112 (16%) Survey A and 76 (15%) Survey B and C respondents. Common responses included expressions of appreciation or inspiration, suggestions for additional research questions, and personal stories.

## Discussion

Following established methodological frameworks and working with diverse interest-holders, including experts by lived experience, we have described priority questions for future dementia and brain health research in Scotland. The questions are broad, and one-single question could support multiple research projects using differing methodologies and approaches. While we present in ‘top ten’ style, all the questions making the top ten list were perceived as priority, and researchers should not only focus on the ‘number one’ research question.

We are now working with funders and commissioners on the dissemination and implementation of these priorities. While there have been previous attempts to define research priorities, this is the first exercise conducted following recent fundamental changes in dementia care (the advent of disease-modifying agents and biomarkers) and the first to specifically collate priorities around brain health.

### Priorities in context

The research priorities identified show some overlap with previous exercises. For example, in an Irish RPSE published in 2023 [30] early diagnosis, prevention, treatment and the needs of caregivers featured in the aggregate ‘top ten’. The last pan-UK RPSE [16], published in 2015, prioritised remaining independent, support for caregivers and caring for people with advanced dementia. The previous Scottish RPSE in 2011 [15] rated early detection as the primary priority.

Our exercise also prioritised novel research questions. As well as all the brain health priorities, priorities around repurposing existing drugs, dementia-friendly care and learning from other countries have not previously been suggested in RPSEs. While all the top ten research questions were perceived as impactful by experts, dementia-related priorities received higher overall impact scores. This may reflect the more established nature of dementia research, or the perceived urgency of disease-related topics compared to preventive brain health.

### Strengths and limitations

We used established methods (ensuring comprehensive and transparent reporting), working in partnership with interest-holders, including participants and responses from diverse groups. However, we recognise that even with targeted recruitment, engagement from certain ethnic groups, young adults and the oldest-old could have been better. Indeed, in post-hoc subgroup analyses, priorities of younger participants appeared to differ from older adults. Our methods will have favoured responses from people comfortable with online materials and may have missed less digitally engaged communities. It is possible other research questions would have been prioritised if other age groups were represented more than in the current RPSE. Other limitations represent the inevitable trade-offs necessary to allow completion in our limited time and funding window. For example, having a substantial reduction in number of participants completing the Delphi surveys compared to survey A could suggest attrition bias that could have been mitigated with more time and resources. Although we strived to make processes as transparent as possible, designing all elements to be fair and objective, RPSEs remain subjective and context-dependent, albeit more objective than traditional paradigms where single expert(s) dictate the research agenda.

### Recommendations

We have collated nationally agreed research priorities that are suitable for informing future research questions and have been prioritised for impact. We are now working with academics, funders and third sector to raise the visibility of these priorities in the anticipation of commissioned research.

Our motivation for a Scotland-specific RPSE was the need for research that is relevant to local context. However, many of our prioritised questions may be relevant to other countries and healthcare systems. We have shown that prioritisation can be conducted at speed and scale, and would encourage other countries to adopt our approach to develop their own priority research agenda. Accepting the limitations in representation of certain groups, our stratification analyses suggest that people aged under 40 years have different priorities. So, given the life course nature of brain health, this specific subgroup could be targeted in future brain health RPSEs.

## Conclusion

It is important for Scotland, a country that has already been pioneering in equitable dementia and brain health research and policy, to allow the public to set the next targets for research. We have shown that robust, national prioritisation can be completed in a timely fashion and our approach can be a blueprint for other countries. We now urge researchers, funders and decision-makers to adopt the top ten priority lists and commit to collaborative research efforts that address them. By doing so, Scotland can continue to lead globally in shaping dementia research that truly reflects societal needs and drives meaningful impact.

## Supplementary Material

Supplementary_materials_afag261

## Data Availability

Data is available from the authors upon reasonable request.
