# Supplementary material for: Scottish priorities for dementia and brain health research—results of a national research priority setting exercise
Source: Age Ageing. 2026 Oct 20;55(10):afag261. doi: 10.1093/ageing/afag261 (PMC13680006; doi:10.1093/ageing/afag261)
Supplement: Supplementary file 1 [file supplementary_materials_afag261.docx]

## **Scottish priorities for dementia and brain health research – results of a national research priority setting exercise**

**SUPPLEMENTARY MATERIAL**

[**Scottish priorities for dementia and brain health research – results of a national research priority setting exercise**	1](#_Toc236916220)

[**Appendix 1: Frameworks**	2](#_Toc236916221)

[Tan et al.^1^ framework	2](#_Toc236916222)

[REPRISE framework	3](#_Toc236916223)

[**Appendix 2: steering board demographics**	6](#_Toc236916224)

[**Appendix 3: CHERRIES**	7](#_Toc236916225)

[**Appendix 4: survey A**	11](#_Toc236916226)

[**Appendix 5: topics excluded because of evidence base**	15](#_Toc236916227)

[**Appendix 6: ranking phase surveys**	15](#_Toc236916228)

[Survey B (including full interim list)	15](#_Toc236916229)

[Survey C (including full short list)	26](#_Toc236916230)

[Survey D	30](#_Toc236916231)

[**Appendix 7: demographics survey D**	36](#_Toc236916232)

[**Appendix 8: interim list themes**	36](#_Toc236916233)

[**Appendix 9: Delphi 1 analysis age groups**	37](#_Toc236916234)

[**References**	38](#_Toc236916235)

## **Appendix 1: Frameworks**

### Tan et al.^1^ framework

Preparatory phase:

- People: steering board with presentation of different interest-holder groups, gender, somewhat regional, less ethnicity diversity than Scotland (although Census 2022 data^2^ stated 85-90% of Scottish people is white). No conflict of interest
- Objectives:

i) To elicit feedback from relevant interest-holders in brain health on the priority areas for brain health and dementia research in Scotland.
ii) To inform a research agenda of funders, policy makers and the wider research community, so that limited resources are spent on research that matters most to the people whom it concerns.
iii) To make a wider public aware of the topic of and research efforts in brain health in Scotland.

- Context: NIHR call for guidance on commissioned funding calls but also making it broader: as Brain Health ARC wants to make Scotland top destination for brain health research; Also ENRICH, NRS Ageing, NRS Scotland and Brain Health Scotland – part of Alzheimer Scotland – were involved. Scotland has National Health Service, just like rest of UK, and mostly government-affiliated institutes are responsible for financing research. There is an ageing population in Scotland (>1mill >65y in Census 2022^2^), with it increase in dementia (estimated 90.000). Life-course approach to maintain brain health and prevent disease has become widely accepted by policy and research bodies. Since 2019^3^, there is a national initiative with accompanying website www.brainhealth.scot. At the same time Scotland’s lay public has low awareness of dementia risk reduction and potential of brain health, according to Brain Health Scotland 2023 survey report about attitudes towards brain health, dementia risk reduction and early detection^4^. There has been the start of Brain Health Services in Aberdeen. It is specifically mentioned in the vision described in Scotland’s 2023 dementia strategy the government also sees the importance of prevention/early detection and how society needs to have an understanding of it^5^. There is no consensus on brain health definition. For this study we want an inclusive scope, but because of the background/set up of our team our context is that of cognitive brain health in older adults. We do include the life course idea of brain health (e.g. research projects like STAR^6^ or cardiovascular risk factors related to brain health) in how we consider the term and what prioritised research topics will be considered for our priority list.
- Interest-holder: professional ((multidisciplinary) clinicians/health care professionals, academics (such as NIHR), third sector (such as Brain Health Coalition UK), government) or lived experience with dementia or mild cognitive impairment (personally, as caregiver) or having interest in brain health (any Scottish citizen could be eligible). Results should be shared with people having special interest in the topic (so through websites of institutes) but also try to make it a national topic as everyone is at risk.
- Evidence: Our recent systematic review showed no prioritisation exercises in brain health anywhere, only dementia (often including prevention). Previous James Lind Alliance (JLA) Priority Setting Partnership’s (PSP) priorities on dementia in UK^7^ were mostly about the theme care, not as much prevention. Survey in Scotland yielded early detection as highest priority^8^.However, interest has tremendously grown on the topic brain health since then. Dementia prioritisation exercises in other countries yield prevention as an important topic. Scotland is small enough to do national priorities and has a vast amount of research networks and funding bodies, mostly governmental, some of them already partnering on other projects and initiatives.

Priority setting phase:

- Criteria: when looking at 2023 Dementia strategy again, participation/inclusion are big principles, this can be further specified into rural and socio-economic inclusion. Also, practicality and independence. Further we can adapt Shah et al WHO criteria for impact/feasibility^9^ (see REPRISE framework below).
- Method: We choose a non-linear approach to data collection of questions, through combining an online survey (integrated some JLA methods, not follow) and results of discussions in a research meeting and the Scottish Dementia Research Consortium yearly conference. After collecting topics, we will collate this in a long list (merging duplicates and categorising by thematic analysis) and it will be checked if any questions should be taken out because they are already answered in literature. Then, we will email this interim list to participants, giving them one week to add any more, in case they were inspired by answers of others. Subsequently, we will conduct a Delphi process through online survey using a formal ranking method (first round scoring (keep, exclude, nothing) followed by latter round(s) forced ranking) of collected questions/priorities. The steering board will discuss final list based on set feasibility criteria, this way the wisdom of the masses is combined with expertise.
- Reporting: using REPRISE guideline^10^, we will make an elaborate report for scientific journals.

Follow-up phase

- Dissemination: to researchers through a scientific paper (with steering board members as group authors) and through conferences (minimally SDRC 2026 annual meeting, rest depending on acceptance of submissions). With funders, third sector charities, and participants we will share a lay-language condensed version of the report, maximum of two pages. To a wider public of interest-holders through website news items of involved organisations (Brain Health ARC/SDRC, Alzheimer Scotland/Brain Health Scotland); a video clip on our social media; look for collaboration with academic dementia blogger Adam Smith; share through CHAIN subgroup dementia mailing list; and we will contact the media team of University of Glasgow once publication comes closer.
- Research: based on a NIHR funding call but will also be presented to relevant other research institutes/funding bodies.
- Evaluation: Opportunity to fill out feedback in surveys and reach out on email. Steering board evaluation meeting.

Sustainability phase

- Institutionalisation: Brain Health ARC for now. Results will be shared with collaboration of NIHR, Alzheimer’s Society, and James Lind Alliance, who are currently planning on another priority-setting partnership about dementia. Otherwise, anticipate new cycle in 5-10 years. With a database search beforehand whether priorities were picked up.
- Peer-learning and sharing: most of it will be reported on in a scientific paper. Steering board members will engage in their research meetings as usual and are representing such exercises, UK broad. FL will return to Netherlands and give presentations about it there. Also, her VU supervisor, who is an important researcher in geriatrics, will read and assess the paper.

### REPRISE framework

| **No** | **REPRISE item** | **Adaptation to our research** |
| --- | --- | --- |
| A. Context and scope | | |
| 1 | Define geographical scope | Scotland |
| 2 | Define health area, field, focus | Brain health and dementia |
| 3 | Define the intended beneficiaries | Everyone at risk for deteriorating brain health |
| 4 | Define the target audience of the priorities | NIHR and other research funding bodies in Brain Health and Dementia (in UK/Scotland). |
| 5 | Identify the research area | All: basic science, health service, public health, clinical, etc |
| 6 | Identify the type of research questions | All allowed: e.g. pathophysiology, interventions, economic evaluation, etc |
| 7 | Define the time frame | 5-10 years |
| B. Governance and team | | |
| 8 | Describe the selection and structure of the leadership and management team | Through TQ’s network in research and policy about dementia/brain health. FL data manager, TQ principal investigator. Rest is consulted when important decisions need to be made. Report number of (online) meetings over course of project time. Members lived experience through Partners in Research. Met three times during the study, one-hour online meetings prepared by FL/TQ (and once core team), in which members were consulted on specific topics and there was room to comment on anything. |
| 9 | Describe the characteristics of the team | Affiliations (main field research/ policy/ clinician/ PLWD), region of Scotland, age, ethnicity (see Appendix 2). |
| 10 | Describe any training or experience relevant to conducting priority setting | TQ involved in JLA exercise, several other research projects; FL did review and quality assessment of priority exercises and attended Cochrane/Plymouth webinar series about research priority setting; LS did a qualitative priority exercise^11^; EL did a quantitative priority exercise^8^ and was part of the JLA PSP^7^; SS participated as expert in nominal group technique Delphi style priority exercise^12^. |
| C. Framework for priority setting | | |
| 11 | State the framework used (if any) | Tan et al^1^, adapting JLA survey and Delphi consensus building |
| D. Stakeholder or participants | | |
| 12 | Define the inclusion criteria for stakeholders involved in priority setting | professional (clinicians/health care professionals, academics, third sector) or lived experience with dementia or mild cognitive impairment (personally, as caregiver) or having interest in brain health (any Scottish citizen could be eligible). |
| 13 | State the strategy or method for identifying and engaging stakeholders | Convenience sample, through institutional websites (Join Dementia Research (JDR), Brain Health ARC, Brain Health Scotland (BHS) by Alzheimer Scotland (AS), The Scottish Dementia Research Consortium (SDRC)). Convenience targeting through: informal network of Asian and Black scientific groups in Scotland; specifically reaching out to non-white volunteers on JDR (as stated in their profile).  Specifically: JDR is a UK-wide platform, and volunteers within Scotland could access the page of this study and show interest and/or access the survey link directly. We also reached out to as many people as we could while the survey was active, starting with the most recently updated profiles. Out of 5 048 Scottish volunteers, 281 directly contacted the researcher team to express an interest and we reached out to 1 680 more, either through phone or email, depending on the profile preferences. Online distribution was done through Bluesky, X, Viva Engage of University of Glasgow, NHS Research Scotland Ageing and the informal networks of steering board members. During the SDRC meeting, a QR-code to the survey was shown during a presentation and people could write down priorities on paper copies.  Participants could fill out their email addresses if they wanted to be involved in later phases.  For the last/fourth survey experts were identified through the executives’ networks of Brain Health ARC, Scottish Dementia Research Consortium, Alzheimer’s Research UK (Scotland), Scottish Cognitive Ageing Network, and members of the steering board for this study. |
| 14 | Indicate the number of participants and/or organizations involved | Number of participants 706 in first, 242 in second, 252 in third, 17 in fourth survey. Number of organisations: six (JDR, Brain health ARC, BHS, the SDRC, AS) |
| 15 | Describe the characteristics of stakeholders | Survey collects interest-holder role, type of living environment, self-identified gender and ethnicity, age group.  For the expert survey slightly different demographics were collected to capture the degree of expertise and representation of scholars throughout Scotland (such as university affiliated with and years of experience in dementia research). |
| 16 | State if reimbursement for participation was provided | No |
| E. Identification and collection of research priorities | | |
| 17 | Describe methods for collecting initial priorities | Previous meeting of TQ and other researchers through discussion, online survey (piloted via Active Voice Research Group of Alzheimer Scotland) where people formulate priorities, SDRC summit through discussion. A collated list will be emailed to participants and give them a week to email more questions if inspired by others. This email also consisted of an explanation of the process and reference to current evidence on the website of Brain Health Scotland. |
| 18 | Describe methods for collating and categorizing priorities | Mixed inductive & deductive thematic analysis in Microsoft Office (Excel and Word). Themes from previous systematic reviews^13, Lunter unpublished^ were adopted (pathology; diagnosis; intervention; support; awareness & education; research methods; system & logistical challenges; equity). |
| 19 | Describe methods and reasons for modifying (removing, adding, reframing) priorities | Removal when: duplicate or represented in other question; put in different grammar than is preferred; only submitted once (exception if considered important enough to keep in by core team). |
| 20 | Describe methods for refining or translating priorities into research topics or questions | By FL during collation process of inductive thematic analysis, discussed with TQ, checked with steering board, iteratively throughout study. |
| 21 | Describe methods for checking whether research questions or topics have been answered | Discussed by FL and TQ, mostly based on the latter’s extensive knowledge of the research field. Checked with steering board by providing list of topics that were excluded on this basis. |
| 22 | Describe number of research questions or topics | Make flow diagram of methodological steps and result in number of questions (see report). |
| F. Prioritization of research topics/questions | | |
| 23 | Describe methods and criteria for prioritizing research topics or questions | Methods: Delphi process in online survey. First time (because it will be many questions) three-point grading (keep, exclude, neutral) of questions presented in categories (that were randomised). Average score yielded the priority order. Participants were also asked to select up to two themes that were most important to them. Round after that: prioritisation through forced ranking (after selecting questions ranking them). Borda count (where questions ranked 1 would get a score of 10, ranked 2 of 9, etc; questions that weren’t chosen to rank would get a score 0) yielded the priority order. Lastly, steering board would finalise top ten based on criteria below.  Criteria (adapted from Shah et al^9^): feasibility of study, burden, novelty, implementation, equity. However, because of split shortening was not necessary anymore. Chose to do an expert survey with these criteria, although they do not influence ranking order. To avoid survey fatigue bias, it was randomised if participants had to appraise brain health or dementia research priorities first. |
| 24 | State the method or treshold for excluding research topics/questions | Cut-off numbers were agreed upon during discussion within a steering group meeting, combining lived experience from those members and professional expertise what is feasible for a Delphi approach. In beginning there will be a threshold set based on number of people (collating interim list, >100 people participating in total), later on number of questions (Delphi 1 yields 50 question-list (+/- 5 or even less if there is a natural divide in prioritisation scores) ; Delphi 2 yields max 20 question-list (per split theme brain health & dementia, if applicable)). There was a natural divide after Delphi 2, so a split was agreed upon (advantages: better visibility of brain health and less bias by interest-holders’ experience with dementia rather than brain health). |
| G. Output | | |
| 25 | State the approach to formulating the research priorities | Format of a research question. We tried to keep the question as short as possible (using similar linking words and formats for similar themed questions), and if a specific element was submitted often, it was added after the question mark between brackets. |
| H. Evaluation and feedback | | |
| 26 | Describe how the process of prioritization was evaluated | Opportunity to fill out feedback in surveys and reach out on email. Steering board evaluation meeting will follow later during follow-up phase. |
| 27 | Describe how priorities were fed back to stakeholders and/or to the public; and how feedback (if received) was addressed and integrated | All participants will receive a short, lay language report/pamphlet of outcomes. There will be news items on websites through which the survey was distributed. |
| I. Implementation | | |
| 28 | Outline the strategy or action plans for implementing priorities | NIHR and other funders will be sent the results. |
| 29 | Describe plans, strategies, or suggestions to evaluate impact | Results will be shared with a collaboration group of NIHR, Alzheimer’s Society, and James Lind Alliance, who are currently planning on another priority-setting partnership about dementia. If this falls through, anticipate new cycle in 5-10 years. With a database search beforehand whether priorities were picked up. |
| J. Funding and conflict of interest | | |
| 30 | State sources of funding | TQ has funding through Brain Health ARC by SFC and besides that SDRC is funded by charity Alzheimer Scotland.  LS has funding through the Global Brain Health Institute, Alzheimer's Association and Alzheimer's Society for my priority-setting project but have no other conflicts of interest.  SM has funding from Dunhill Medical Trust.  SS has funding from BBSRC, NIHR Team Science, Legal and General for ACRC, CSO for ENRICH, but no conflict of interest.  EL has no conflict of interest.  TR’s network is funded by Scottish government through Chief Scientist Office.  AM is employed by Alzheimer Scotland. |
| 31 | Declare any conflicts or competing interests | None |

## **Appendix 2: steering board demographics**

| **Name (gender) role** | **Interest-holder role** | **Affiliations and location** | **Age group** | **Ethnicity** |
| --- | --- | --- | --- | --- |
| **Prof Quinn, Terence J. (m)** Chief investigator & chair & core team | R/C/P/L | University of Glasgow researcher on stroke / dementia / brain health / research methodology, professor in cardiovascular ageing; consultant geriatrics & stroke; Chair of Brain Health ARC | 40-49 years | White |
| **Lunter, Fenna M.A. (f)** Data coordinator & lead & core team | R/C | Affiliate researcher University of Glasgow; final year medical student Vrije Universiteit Amsterdam (NL) | 20-29 years | White |
| **Henry, Winnie (f)** Core team & consulting member | L/R | Lived experience as caregiver for spouse, has since gotten involved in multiple research projects and in Partners in Research. Retired psychotherapist. Situated in North Ayrshire region. | 80-89 years | White |
| **Ross, David (m)** Core team & consulting member | L/R | Lived experience as caregiver for multiple family members. Involved in Partners in Research. Situated in Glasgow area. | 70-79 years | White |
| **Kerr, Jacqui (f)** Consulting member | R/C/P | Network Manager Neuroprogressive & Dementia Network in Dundee; Nurse in Elderly Mental Health; Research portfolio on neurodegenerative disease | 50-59 years | White |
| **Dr Law, Emma (f)** Consulting member | R/C/P | Strategic Manager Neuroprogressive and Dementia Research Network, co-chair ENRICH; research portfolio on research delivery in dementia; mental health nurse clinical experience. | 60-69 years | White |
| **Dr Makin, Stephen (m)** Consulting member | C/R | Consultant geriatrics Wick; research experience in Aberdeen on stroke, ageing and rural health | 40-49 years | White |
| **McKean, Alison (f)** Consulting member | P/C | Executive lead for Brain Health and Research at Alzheimer Scotland; Occupational Therapist in dementia and mental health field. | 40-49 years | White |
| **Dr Russ, Tom C. (m)** Consulting member | R/C | Consultant old age psychiatry Edinburgh; research experience in dementia epidemiology | 40-49 years | White |
| **Dr Stirland, Lucy E. (f)** Consulting member | R/C | Finished a priority exercise in Scotland at University of Edinburgh in 2025; research portfolio on multimorbidity and dementia; Old age psychiatrist | 30-39 years | White |
| **Prof Shenkin, Susan D. (f)** Consulting member | R/C/P | Professor of Healthcare for Older people at University of Edinburgh, research on care homes, co-chair ENRICH; consultant geriatrics | 50-59 years | White |

**Affiliations of steering board members** m = male; f = female; R = researcher; C = clinician; P = policy/third sector; L = lived experience (living with or caring for someone with dementia). Shown in alphabetical order per category of involvement.

## **Appendix 3: CHERRIES**

Table retrieved from original paper^14^.

|  | **Checklist for Reporting Results of Internet E-Surveys (CHERRIES)** | | |
| --- | --- | --- | --- |
| ***Item Category*** | ***Checklist Item*** | ***Explanation*** | ***Our study*** |
| **Design** | | | |
|  | Describe survey design | Describe target population, sample frame. Is the sample a convenience sample? (In “open” surveys this is most likely.) | Convenience sample, >100. |
| **IRB (Institutional Review Board) approval and informed consent process** | | | |
|  | IRB approval | Mention whether the study has been approved by an IRB. | Yes, the survey is part of a research project that was approved by University of Glasgow: College of Medical, Veterinary & Life Sciences Ethics Committee, project no 200240255 |
|  | Informed consent | Describe the informed consent process. Where were the participants told the length of time of the survey, which data were stored and where and for how long, who the investigator was, and the purpose of the study? | There was a participant information sheet, a privacy notice and consent form available through either email or embedded within one of the first pages of the survey. An indication was given in the email accompanying an invite to the survey. |
|  | Data protection | If any personal information was collected or stored, describe what mechanisms were used to protect unauthorized access. | Contact information on university secured networks, will be deleted after the end of the project. Answers within the survey are not retraceable. |
| **Development and pre-testing** | | | |
|  | Development and testing | State how the survey was developed, including whether the usability and technical functionality of the electronic questionnaire had been tested before fielding the questionnaire. | Several draft versions were made, incorporating feedback from the steering board and piloting (first survey on Active Voices group). Technical functionality was already double checked with peers before airing as a pilot. |
| **Recruitment process and description of the sample having access to the questionnaire** | | | |
|  | Open survey versus closed survey | An “open survey” is a survey open for each visitor of a site, while a closed survey is only open to a sample which the investigator knows (password-protected survey). | Open |
|  | Contact mode | Indicate whether or not the initial contact with the potential participants was made on the Internet. (Investigators may also send out questionnaires by mail and allow for Web-based data entry.) | Some were through internet first (websites or JDR platform) and some participants were first reached through email. |
|  | Advertising the survey | How/where was the survey announced or advertised? Some examples are offline media (newspapers), or online (mailing lists – If yes, which ones?) or banner ads (Where were these banner ads posted and what did they look like?). It is important to know the wording of the announcement as it will heavily influence who chooses to participate. Ideally the survey announcement should be published as an appendix. | For survey A-C through institutional websites of Join Dementia Research (JDR), Brain Health ARC, Brain Health Scotland (BHS) by Alzheimer Scotland (AS), The Scottish Dementia Research Consortium (SDRC). We created a BlueSkye and X account to post survey links on, which could be reposted on these platforms. Moreover, dissemination happened through the network of steering board members. Specific subpopulation targeting as part of iterative inclusion process (based on demographics, such as ethnicity and gender) of the sample so far, comparing to Scotland’s population.  Survey D was still an anonymous link without password but specific experts in dementia research were invited through email. |
| **Survey administration** | | | |
|  | Web/E-mail | State the type of e-survey (eg, one posted on a Web site, or one sent out through e-mail). If it is an e-mail survey, were the responses entered manually into a database, or was there an automatic method for capturing responses? | Automatic response capturing through Qualtrics. |
|  | Context | Describe the Web site (for mailing list/newsgroup) in which the survey was posted. What is the Web site about, who is visiting it, what are visitors normally looking for? Discuss to what degree the content of the Web site could pre-select the sample or influence the results. For example, a survey about vaccination on a anti-immunization Web site will have different results from a Web survey conducted on a government Web site | Institutional websites focused on knowledge sharing and support of non-profit (research) projects. Most visitors might have an above average educational level, and are probably already involved or interested in similar (research) projects. |
|  | Mandatory/ voluntary | Was it a mandatory survey to be filled in by every visitor who wanted to enter the Web site, or was it a voluntary survey? | Voluntary |
|  | Incentives | Were any incentives offered (eg, monetary, prizes, or non-monetary incentives such as an offer to provide the survey results)? | No monetary incentives. The result of the entire study (where the surveys are part of) was announced to be shared through similar media as recruitment (websites). Those whose email address was known will receive a short report/pamphlet with results. |
|  | Time/Date | In what timeframe were the data collected? | All three public surveys (A-C) in chronological order: April and July 2025. Expert survey (D) in July 2025. |
|  | Randomization of items or questionnaires | To prevent biases items can be randomized or alternated. | Survey A, collecting topics, had a fixed order.  The first Delphi survey (B) had the themes that had to be scored randomised. The second Delphi (C) had no randomised questions. And D, the expert survey, had the blocks with dementia or brain health priorities randomised. |
|  | Adaptive questioning | Use adaptive questioning (certain items, or only conditionally displayed based on responses to other items) to reduce number and complexity of the questions. | Yes, in survey A only when a prioritised research topic/question was filled out, a next box appeared, up to 10 questions total. In surveys B-D an explanation of brain health was only shown if pointed out someone had not taken part before or wanted to see it again. In survey C only the 10 questions that were picked out of the list of 50 were subsequently shown to drop-and-rank. |
|  | Number of Items | What was the number of questionnaire items per page? The number of items is an important factor for the completion rate. | There was a maximum of two questions per page, but most often only one. Some questions did consist of more Likert scale elements. The maximum in Survey B was 11 (questions in subtheme III.A) per page and in Survey D 50 (5 criteria for 10 priorities). |
|  | Number of screens (pages) | Over how many pages was the questionnaire distributed? The number of items is an important factor for the completion rate. | In survey A, there were minimally 15 pages to go through, up to 24 if all 10 topics were filled out. Survey B had 55 pages, people who did not see the explanation again had 54. Survey C had 20 pages (or 19 without explanation). Survey D had 38 pages (or 37 without explanation). |
|  | Completeness check | It is technically possible to do consistency or completeness checks before the questionnaire is submitted. Was this done, and if “yes”, how (usually JAVAScript)? An alternative is to check for completeness after the questionnaire has been submitted (and highlight mandatory items). If this has been done, it should be reported. All items should provide a non-response option such as “not applicable” or “rather not say”, and selection of one response option should be enforced. | In survey A, all demographic questions and the first prioritised research topic/question were mandatory. In survey B-D all questions but the feedback question were mandatory. This was done in Qualtrics by adding the Requirement “Force response” for each question. The demographic questions all had a ‘Prefer not to say’ or ‘Other: …’ option. |
|  | Review step | State whether respondents were able to review and change their answers (eg, through a Back button or a Review step which displays a summary of the responses and asks the respondents if they are correct). | A back button was available, and the last page stated a reminder that this was the last time to change any answers. |
| **Response rates** | | | |
|  | Unique site visitor | If you provide view rates or participation rates, you need to define how you determined a unique visitor. There are different techniques available, based on IP addresses or cookies or both. | n/a |
|  | View rate (Ratio of unique survey visitors/unique site visitors) | Requires counting unique visitors to the first page of the survey, divided by the number of unique site visitors (not page views!). It is not unusual to have view rates of less than 0.1 % if the survey is voluntary. | n/a |
|  | Participation rate (Ratio of unique visitors who agreed to participate/unique first survey page visitors) | Count the unique number of people who filled in the first survey page (or agreed to participate, for example by checking a checkbox), divided by visitors who visit the first page of the survey (or the informed consents page, if present). This can also be called “recruitment” rate. | n/a |
|  | Completion rate (Ratio of users who finished the survey/users who agreed to participate) | The number of people submitting the last questionnaire page, divided by the number of people who agreed to participate (or submitted the first survey page). This is only relevant if there is a separate “informed consent” page or if the survey goes over several pages. This is a measure for attrition. Note that “completion” can involve leaving questionnaire items blank. This is not a measure for how completely questionnaires were filled in. (If you need a measure for this, use the word “completeness rate”.) | In Qualtrics you can see completed and ‘in progress’ numbers. If the completed is divided by all who opened the survey (completed + in progress), NB we cannot see if these are the same people:  Survey A: 706/1158 (61%)  Survey B: 242/361 (67%)  Survey C: 252/351 (72%)  Survey D: 17/33 (52%) |
| **Preventing multiple entries from the same individual** | | | |
|  | Cookies used | Indicate whether cookies were used to assign a unique user identifier to each client computer. If so, mention the page on which the cookie was set and read, and how long the cookie was valid. Were duplicate entries avoided by preventing users access to the survey twice; or were duplicate database entries having the same user ID eliminated before analysis? In the latter case, which entries were kept for analysis (eg, the first entry or the most recent)? | Yes, Qualtrics requires this to function. Also, people were able to come back to the survey later. |
|  | IP check | Indicate whether the IP address of the client computer was used to identify potential duplicate entries from the same user. If so, mention the period of time for which no two entries from the same IP address were allowed (eg, 24 hours). Were duplicate entries avoided by preventing users with the same IP address access to the survey twice; or were duplicate database entries having the same IP address within a given period of time eliminated before analysis? If the latter, which entries were kept for analysis (eg, the first entry or the most recent)? | Yes, by default in Qualtrics. However, the data was completely anonymized, so the IP address was not recorded nor available to the researchers. |
|  | Log file analysis | Indicate whether other techniques to analyze the log file for identification of multiple entries were used. If so, please describe. | The Qualtrics “prevent multiple submissions” option was on (flagging the double responses, to be checked by the researcher and the one being identified as the correct one was kept), as was the “RelevantID” option (to prevent fraudulent responses locking people out of follow-up survey sessions, with a resetting after 1 week). However, no submissions were excluded based on this. |
|  | Registration | In “closed” (non-open) surveys, users need to login first and it is easier to prevent duplicate entries from the same user. Describe how this was done. For example, was the survey never displayed a second time once the user had filled it in, or was the username stored together with the survey results and later eliminated? If the latter, which entries were kept for analysis (eg, the first entry or the most recent)? | See above: flagging the double responses, to be checked by the researcher and the one being identified as the correct one (based on what was filled out seemed intentional) was kept. If no difference was to be seen, the first one was kept. |
| **Analysis** | | | |
|  | Handling of incomplete questionnaires | Were only completed questionnaires analyzed? Were questionnaires which terminated early (where, for example, users did not go through all questionnaire pages) also analyzed? | Only completed surveys were included. |
|  | Questionnaires submitted with an atypical timestamp | Some investigators may measure the time people needed to fill in a questionnaire and exclude questionnaires that were submitted too soon. Specify the timeframe that was used as a cut-off point, and describe how this point was determined. | Not analysed |
|  | Statistical correction | Indicate whether any methods such as weighting of items or propensity scores have been used to adjust for the non-representative sample; if so, please describe the methods. | No |

## **Appendix 4: survey A**

Q1 Thank you for participating in this survey. We highly value your opinion.

Our aim is to find out which are the most important questions for future brain health research. In this project we are interested in the views of adults living in Scotland.

We want to hear the opinions of people like you, who have an interest in dementia and brain health. This will make sure that research addresses topics that matter to the people affected.

At the end of this project, we will create a top ten list of research priorities for brain health in Scotland. This survey is one of the first steps.

Q2 In this survey we will ask you to list the brain health questions that you think are important, and that could be answered by research.

We will also collect demographic details. This is to find out more about who has completed the survey. It will not be possible to identify any individual respondent from this survey.

If at any point you feel like you do not want to take part anymore, you are free to stop the survey. Your participation is entirely voluntary. Also, it is possible to take a break and finish the survey later.

Q27 Please find full study information and the privacy notice below. You can also find the terms that make up your consent, if you agree to participate. The links will open in new tabs.

1. Participant information sheet surveys 310325

2. Privacy notice surveys 310325

3. Consent form surveys 310325

o	I declare that I am 18 years or older (4)

Q28 and

o	I have read the terms as described in the documents above and agree to participate in this study (1)

Q3 Thank you! Your consent has been registered.

As filling in online surveys may be challenging, feel free to ask someone to assist you.

If you would rather give your views in another way, please email. Then we can explore what fits best, such as a phone appointment or printed survey by post.

Thanks again and let's get started!

Q18 First, we want to learn a little more about you. This way, we can check how well the people of Scotland are represented in our survey.

Q19 What best describes your role?

o	Person living with dementia (1)

o	Person living with mild cognitive impairment (2)

o	Caregiver (3)

o	Former caregiver (4)

o	Researcher (5)

o	Clinician (doctor, nurse, paramedic, professional caretaker, etc) (6)

o	Third sector (policy, charity, etc) (7)

o	Scottish citizen with interest in brain health (8)

o	Other, that is: (9) __________________________________________________

Q20 In what type of location are you currently living?

o	Large city (1)

o	Suburb near a large city (2)

o	Small city or town (3)

o	Rural area (4)

o	Other (5)

o	Prefer not to say (6)

Q21 What gender do you self-identify with?

o	Male (1)

o	Female (2)

o	Non-binary / third gender (3)

o	Prefer not to say (4)

Q25 Which ethnicity do you identify with?

o	White (1)

o	Mixed or multiple (2)

o	Asian, Asian Scottish or Asian British (3)

o	Caribbean or Black (4)

o	Other (5)

o	Prefer not to say (6)

Q22 What age group do you fall in? If you are under 18, unfortunately you cannot participate in this survey and we kindly ask you to close this tab.

o	18-29 years old (1)

o	30-39 years old (2)

o	40-49 years old (3)

o	50-59 years old (4)

o	60-69 years old (5)

o	70-79 years old (6)

o	80-89 years old (7)

o	90-99 years old (8)

o	>100 years old (9)

o	Prefer not to say (10)

Q29 How did you find out about this survey?

o	Website news item (e.g. Brain Health ARC website) (1)

o	Newsletter (e.g. Brain Health Scotland) (2)

o	Forwarded email from an acquaintance (8)

o	Social media (like X or Bluesky) (3)

o	Join Dementia Research platform (5)

o	A pamphlet / presentation slide (e.g. SDRC conference) (6)

o	Other: (7) __________________________________________________

o	Prefer not to say (9)

Q26 Thank you. We now go on to the second part of the survey.

Q4	Quick background

Before we ask you to fill out your prioritised topics for brain health research, we want to give you a short overview of what we mean by brain health.

There are many definitions of brain health. All definitions agree that brain health is more than just absence of diseases like dementia. Brain health means keeping your brain function at the highest level possible, for as long as possible. While many brain diseases are seen in older adults, brain health is important throughout your whole life.

In this study, with brain health we also mean the state before dementia, so in other words preventing dementia.

Recent research has identified factors that can cause poor brain health. These include poor sleep, air pollution and limited access to education. It is estimated that up to 40% dementia diagnoses worldwide could be prevented by attending to these factors.

There may be other factors that are bad for brain health, or can protect brain health. Research could help identify these factors. Research could also describe how to reduce the problems from factors such as poor sleep.

Q5 It is not necessary for participating in this survey, but if you want to read more about brain health go to:

- Brain Health Scotland

- World Health Organization

- Brain Health ARC

- Scientific article about known risk factors by Livingston et al

Q6 You may have responded to the invitation to participate as you are a person living with dementia, or a care partner of someone with dementia. If you have research questions around dementia, we want you to share them. However, our brain health focus means we do not have to limit our thoughts to dementia.

You are not obliged to use this, but the research themes listed in this document (opening in new tab) might help you generate your own ideas about research topics for this survey. You can also find previous research priorities for dementia here (published in 2013), if you want.

Q7 Give yourself time to think. What is important to you, and the people you care about?

What kind of research questions would you like to see answered? Specific topics could work, but broad questions are allowed as well. Don't worry about format or grammar, it is the idea we are interested in. No idea is too silly.

Here are some examples of potential questions.

*"How much air pollution is too much when it comes to protecting our brains from dementia?"*

*"Can listening to music help reduce stress and keep the brain healthy for working age people?"*

*"How can local community groups help people build habits that are good for their brain?"*

*"How can Scottish researchers best keep an overview of international research?"*

If you want to go back to the explanation about themes and brain health, you can by clicking the arrow ← below once or twice. You get the opportunity to fill out up to 10 questions, but fewer is also okay. Then just leave the next question blank.

1. Question about brain health you think should be prioritised by research:

________________________________________________________________

Q8 You get the opportunity to fill out up to 10 questions, but fewer is also okay. Then just leave the next question blank.

2. Question about brain health you think should be prioritised by research:

________________________________________________________________

Q9 You get the opportunity to fill out up to 10 questions, but fewer is also okay. Then just leave the next question blank.

3. Question about brain health you think should be prioritised by research:

________________________________________________________________

Q10 You get the opportunity to fill out up to 10 questions, but fewer is also okay. Then just leave the next question blank.

4. Question about brain health you think should be prioritised by research:

________________________________________________________________

Q11 You get the opportunity to fill out up to 10 questions, but fewer is also okay. Then just leave the next question blank.

5. Question about brain health you think should be prioritised by research:

________________________________________________________________

Q12 You get the opportunity to fill out up to 10 questions, but fewer is also okay. Then just leave the next question blank.

6. Question about brain health you think should be prioritised by research:

________________________________________________________________

Q13 You get the opportunity to fill out up to 10 questions, but fewer is also okay. Then just leave the next question blank.

7. Question about brain health you think should be prioritised by research:

________________________________________________________________

Q14 You get the opportunity to fill out up to 10 questions, but fewer is also okay. Then just leave the next question blank.

8. Question about brain health you think should be prioritised by research:

________________________________________________________________

Q15 You get the opportunity to fill out up to 10 questions, but fewer is also okay. Then just leave the next question blank.

9. Question about brain health you think should be prioritised by research:

________________________________________________________________

Q16 You get the opportunity to fill out up to 10 questions, but fewer is also okay. Then just leave the next question blank.

10. Question about brain health you think should be prioritised by research:

________________________________________________________________

Q23 That was it! Is there anything you would like to add or change in your answers? Now is the last time you can still go back using the arrow ← below.

If there is any feedback (on either this survey or the prioritisation research in general), feel free to write it here:

________________________________________________________________

Q24 Looking forward

The responses will be collected and after editing you will be invited to take part in the next round: prioritising collected questions. Study end results will be shared through the same platforms used for finding participants.

Can we contact you for upcoming surveys to rank the questions that were submitted? We would really appreciate it if we can. You would further contribute to setting the top 10 research priorities on brain health for Scotland.

If yes, please fill in your email address below. This personal information will be kept at a secured university network and will be deleted at the end of the project.

This is the last page of this survey.

o	Email address (1) __________________________________________________

## **Appendix 5: topics excluded because of evidence base**

- alcohol
- smoking
- vascular risk factors (interventions)
- sex life
- early life education
- multilingualism
- general anaesthetics
- hearing decline
- vision decline
- depression/mood disorders
- can we slow down/prevent
- reasons for rise prevalence
- current pharmacological options
- communication intervention (speech therapy)
- evaluation current policy strategies (Brain Health Services, national dementia strategy, role GPs, NHS standard care and triangle of care)

## **Appendix 6: ranking phase surveys**

### Survey B (including full interim list)

Q1 Thank you for participating in this second survey. We highly value your opinion. Our aim is to find out which are the most important questions for future brain health research. In this project we are interested in the views of adults living in Scotland.

We want to hear the opinions of people like you, who have an interest in dementia and brain health. This will make sure that research addresses topics that matter to the people affected.

Q2 In this survey we will ask you to grade the importance of brain health research questions that we collected in the previous survey. These were collated into a long list, which is a step closer to establishing the Scottish top 10 research priorities for brain health. This top 10 will be given to research funding bodies among others.

We will also collect demographic details. This is to find out more about who has completed the survey. It will not be possible to identify any individual respondent from this survey. If at any point you feel like you do not want to take part anymore, you are free to top the survey. Your participation is entirely voluntary. Also, it is possible to take a break and finish the survey later.

Q27 Please find full study information and the privacy notice below. You can also find the terms that make up your consent, if you agree to participate. Ticking the boxes below suffices as consent, you don't need to email anything to us.

All documents are the same as the previous survey. The links will open in new tabs.

1. Participant information sheet surveys 310325

2. Privacy notice surveys 310325

3. Consent form surveys 310325

o	I declare that I am 18 years or older (4)

Q28 and

o	I have read the terms as described in the documents above and agree to participate in this study (1)

Q3 Thank you! Your consent has been registered. As filling in online surveys may be challenging, feel free to ask someone to assist you. If you would rather give your views in another way, please email. Then we can explore what fits best, such as a phone appointment or printed survey by post. Thanks again and let's get started!

Q18 First, we need to collect a little information about you again. This way, we can check how well the people of Scotland are represented in our survey.

Q19 What best describes your role?

o	Person living with dementia (1)

o	Person living with mild cognitive impairment (2)

o	Caregiver (3)

o	Former caregiver (4)

o	Researcher (5)

o	Clinician (doctor, nurse, paramedic, professional caretaker, allied health professional, etc) (6)

o	Third sector (policy, charity, etc) (7)

o	Scottish citizen with interest in brain health (for example because you have family members with dementia) (8)

o	Other, that is: (9) __________________________________________________

Q20 In what type of location are you currently living?

o	Large city (1)

o	Suburb near a large city (2)

o	Small city or town (3)

o	Rural area (4)

o	Other (5)

o	Prefer not to say (6)

Q21 What gender do you self-identify with?

o	Male (1)

o	Female (2)

o	Non-binary / third gender (3)

o	Prefer not to say (4)

Q25 Which ethnicity do you identify with?

o	White (1)

o	Mixed or multiple (2)

o	Asian, Asian Scottish or Asian British (3)

o	Caribbean or Black (4)

o	Other (5)

o	Prefer not to say (6)

Q22 What age group do you fall in? If you are under 18, unfortunately you cannot participate in this survey and we kindly ask you to close this tab.

o	18-29 years old (1)

o	30-39 years old (2)

o	40-49 years old (3)

o	50-59 years old (4)

o	60-69 years old (5)

o	70-79 years old (6)

o	80-89 years old (7)

o	90-99 years old (8)

o	>100 years old (9)

o	Prefer not to say (10)

Q32 Did you participate in the previous survey, which collected research topics? If not, you will be redirected to a page with an explanation about the concept of brain health as we introduced it in the first survey.

o	Yes (1)

o	Yes, but i would like to see the explanation again (2)

o	No (4)

Q34 Quick background There are many definitions of brain health. All definitions agree that brain health is more than just absence of diseases like dementia. Brain health means keeping your brain function at the highest level possible, for as long as possible. While many brain diseases are seen in older adults, brain health is important throughout your whole life. Recent research has identified factors that can cause poor brain health. These include poor sleep, air pollution and limited access to education. It is estimated that up to 40% dementia diagnoses worldwide could be prevented by attending to these factors. There may be other factors that are bad for brain health, or can protect brain health. Research could help identify these factors. Research could also describe how to reduce the problems from factors such as poor sleep.

You may have responded to the invitation to participate as you are a person living with dementia, or a care partner of someone with dementia. This is also included in this study. However, our brain health focus means we do not have to limit our thoughts to dementia but cover the whole life course spectrum of (maintaining and declining) brain health.

Q82 This priority setting exercise is about both brain health and dementia.

Because brain health can be a confusing concept Dr Tom Russ, one of the members of our steering group, will now explain what is in and what is out when we talk about brain health in this study. Click here if you want to read it instead of watch the video

Q7 Explaining the current interim priority list We are currently in the ranking phase of our study. Eventually we want to end up with a top 10 list of research priorities for brain health. We build consensus through multiple rounds of ranking.

This round we will do the ranking by grading the importance of each item. This will result in a shorter list of priorities and the next survey you will be asked to put them in a preferred ranking order.

After collecting all 2676 research topics people submitted in the previous survey, we collated this in a long list. If you want to know more about how we did this, click here. Later, you were asked to submit any last ideas. The whole process resulted in a list of 142 questions, categorised in 8 themes with 34 subthemes.

Q44 Set up of this second survey Because this is quite a long list, we will start the ranking process easily. We kindly ask you to go through the questions and say if you think this question should be:

•	OUT of the list, because it is less important to you;

•	NEUTRAL, because you don't mind if it is out or in;

•	IN the list running for top 10, because it is such a priority for you.

The topics will be shown in themes, with the most popular topics on top. The themes are in randomised order to prevent bias.

We want to remind you that you can enter the survey using the same link later again in case you want to take a break. Watch the closing date of the survey, though. When piloting the survey, people took 5 minimum to 20 minutes maximum to complete this survey.

When asked if a research question should be 'out', 'neutral' or 'in': don't overthink it and choose what you want to prioritise. In which order will be something for later surveys.

If you don't know what a question means, we encourage you to choose 'neutral' and let us know in the feedback section at the end of the survey

Q33 We categorised the long list of questions into a theme it fits most. Before we start with the specific research topics, we want to ask you one overview question.

Which of the themes do you find the most important to be prioritised in brain health research? You can select up to two options.

▢	Pathology: understanding the biology of dementia or mechanisms that influence brain health (1)

▢	Diagnosis: identifying who is at risk, and developing tests to help make a diagnosis (2)

▢	Interventions: any treatment (medication, programmes, etc) for individuals or bigger groups influencing dementia (risk) (3)

▢	Support: supporting people with dementia, or at risk of dementia, in daily life (4)

▢	Awareness & Education: how to inform different groups and societal values (5)

▢	Research Methods: to improve the design, conduct, reporting and implementation of research (6)

▢	System & Logistics: designing and optimising systems and logistical pathways (7)

▢	Equity: fair and inclusive dementia care and research (8)

Q35 Now we will start with the grading of topics. There are 8 blocks of themes that will be randomised, each with subthemes, which each have questions.

You can go back and forth within a block using the arrows ← below, but not after you left and entered the next block. We will point out what is the last subtheme of each block.

All questions are important but to focus research efforts, try to think what is MOST important to you

Q38 Pathology Subtheme A-G	A. Lifestyle factors

|  | Take OUT of list (1) | Neutral (2) | Keep IN list (3) |
| --- | --- | --- | --- |
| 1. What is the role of **screen time and social media** in brain health? (including the amount of information offered, type of content and social effects of it) (1) |  |  |  |
| 2. What is the role of **psychoactive drugs** – like benzodiazepines, MDMA, ketamine, etc – in brain health? (including amount, different types, frequency and long-term effects) (2) |  |  |  |
| 3. What is the role of **vaping** in brain health? (during different stages of the life course and long-term effects) (3) |  |  |  |
| 4. What is the role of the **gut microbiome** in brain health? (4) |  |  |  |
| 5. What is the role of **working life factors** in brain health? (including type of occupation and retirement age) (5) |  |  |  |

Q39 Pathology B. Environmental factors

|  | Take OUT of list (1) | Neutral (2) | Keep IN list (3) |
| --- | --- | --- | --- |
| 1. What is the role of **synthetics in consumer products** - like food additives, microplastics and pesticides - in brain health? (1) |  |  |  |
| 2. What is the role of **air and water quality** in brain health? (including pollution sources, threshold for effect and mechanism) (2) |  |  |  |
| 3. Are there **geographical factors** at play in brain health? (such as living in an urban area, near the sea or in specific countries) (3) |  |  |  |
| 4. What is the role of **background noise** in brain health? (including noise pollution) (8) |  |  |  |
| 5. How important is being **outdoors** for brain health? (doing what activities and what amount) (9) |  |  |  |
| 6. What is the impact of **climate change** on brain health? (10) |  |  |  |
| 7. What is the role of certain types of **radiation** in the environment - like microwaves, electricity pylons, Wi-Fi signals and Chernobyl related - in brain health? (11) |  |  |  |
| 8. Do **weather conditions** – like seasons, sunshine – influence brain health and/or dementia symptoms? (12) |  |  |  |

Q58 Pathology C. Psychological factors

|  | Take OUT of list (1) | Neutral (2) | Keep IN list (3) |
| --- | --- | --- | --- |
| 1. What is the role of **social connectedness** in brain health? (including social isolation, owning a pet, receiving kindness) (1) |  |  |  |
| 2. What is the role of **stress and anxiety** in brain health? (including different sources, amounts and length of exposure) (2) |  |  |  |
| 3. What is the role of someone’s **attitude towards life** - like coping and resilience, character traits, optimism, sense of purpose - in brain health? (3) |  |  |  |
| 4. What is the role of (childhood) **trauma** and post-traumatic stress disorder (**PTSD**) in brain health? (4) |  |  |  |
| 5. What is the role of **attachment disorders** in brain health? (5) |  |  |  |
| 6. What is the impact of **being a caregiver** for someone with dementia on their own brain health? (6) |  |  |  |

Q59 Pathology D. Evoked by health care

|  | Take OUT of list (1) | Neutral (2) | Keep IN list (3) |
| --- | --- | --- | --- |
| 1. What is the role of commonly prescribed **medication use** in brain health? (including proton pump inhibitors, antidepressants, statins etc) (1) |  |  |  |
| 2. Do **radiological examinations** – like CT-scans of the brain – have an impact on brain health? (2) |  |  |  |

Q60 Pathology E. Biological development

|  | Take OUT of list (1) | Neutral (2) | Keep IN list (3) |
| --- | --- | --- | --- |
| 1. What is the role of **female hormones** and its fluctuations through life in brain health? (including menopause, menstrual cycle and hormone replacement therapy (including for transpeople)) (1) |  |  |  |
| 2. What is the role of **neurodivergence**, specifically ADHD and autism, in brain health? (including similarities in brain structure) (2) |  |  |  |
| 3. What is the role of **earliest life events** – in the womb, during birth and as a baby – in brain health? (3) |  |  |  |

Q61 Pathology F. Other health conditions as risk factor

|  | Take OUT of list (1) | Neutral (2) | Keep IN list (3) |
| --- | --- | --- | --- |
| 1. What is the role of **long-term health conditions** – like heart disease, diabetes, auto-immune disease etc – in brain health? (1) |  |  |  |
| 2. What is the role of differing forms and severities of **traumatic brain injury** in brain health? (2) |  |  |  |
| 3. What impact does a **pandemic** have on brain health? (including factors as isolation, vaccines and viral exposure) (3) |  |  |  |
| 4. What is the role of **infectious disease** in brain health? (including viral infections such as herpes) (13) |  |  |  |
| 5. What is the role of **oral health** in brain health? (14) |  |  |  |

Q40 Pathology *This is the last subtheme of this theme, so after this page you cannot change your answers.* G. Disease course

|  | Take OUT of list (1) | Neutral (2) | Keep IN list (3) |
| --- | --- | --- | --- |
| 1. **Why do some** people – especially those with high genetic or other risk on paper – **keep good brain health** in reality? (including brain resilience) (1) |  |  |  |
| 2. **How much** can people – especially those with high genetic or other risk for dementia – **reduce** their **risk**? (2) |  |  |  |
| 3. How can we **explain** various **behaviours** seen in **advanced dementia** – like wandering and fluctuations throughout the day? (9) |  |  |  |
| 4. Is there a **crucial age** for determination of brain health? (10) |  |  |  |
| 5. How can we fully understand the **biology of dementia** (subtypes)? (11) |  |  |  |
| 6. Which is the **most important factor** contributing to brain health? (12) |  |  |  |
| 7. Is there a **difference in progress** of cognitive decline between those living at **home** with support versus those living in **care homes**? (13) |  |  |  |

Q55 Diagnosis *Subtheme A-C*	A. Early testing

|  | Take OUT of list (1) | Neutral (2) | Keep IN list (3) |
| --- | --- | --- | --- |
| 1. What are ways to **diagnose dementia earlier** than in current practice? (including identifying key early symptoms and biomarkers) (1) |  |  |  |
| 2. Does an early diagnosis of dementia actually **benefit** the person and their family, considering currently available treatments? (2) |  |  |  |
| 3. What are the benefits and limitations of **screening for dementia** in high-risk groups or brain health in the entire population? (3) |  |  |  |
| 4. Is it possible to **predict poor brain health**, and if so, what are the benefits and downsides of this? (4) |  |  |  |
| 5. What are **requirements for the implementation** of early testing for dementia? (including cost, acceptability, interpreting test results in different populations and ensuring accessibility in NHS) (5) |  |  |  |
| 6. Can a tool be developed to **self-assess** brain health or dementia? (risk, diagnosis, or progression) (6) |  |  |  |
| 7. What are the benefits and limitations of **screening for modifiable brain health risk factors** – like hearing decline? (7) |  |  |  |

Q56 Diagnosis B. Differentiating diagnoses

|  | Take OUT of list (1) | Neutral (2) | Keep IN list (3) |
| --- | --- | --- | --- |
| 1. What is considered **normal ageing** and what is the **threshold for poor brain health**? (1) |  |  |  |
| 2. How can we improve **differentiation between dementia and other conditions** with overlapping symptoms? (like ADHD, depression, brain fog during perimenopause, etc) (2) |  |  |  |
| 3. How can we improve **differentiation between subtypes of dementia** and thereby specificity of diagnosis? (3) |  |  |  |

Q57 Diagnosis *This is the last subtheme of this theme, so after this page you cannot change your answers.* C. Current testing options

|  | Take OUT of list (1) | Neutral (2) | Keep IN list (3) |
| --- | --- | --- | --- |
| 1. Considering accuracy and acceptability, what is the **best way to measure cognitive function** in the Scottish context – like pencil and paper tests, questionnaires or computers? (1) |  |  |  |
| 2. How can **brain scans** best be used in brain health care? (including predicting dementia risk and following brain changes over time) (2) |  |  |  |

Q41 Interventions Subtheme A-C	A. Lifestyle

|  | Take OUT of list (1) | Neutral (2) | Keep IN list (3) |
| --- | --- | --- | --- |
| 1. What is the role of **diet** in brain health and how can we intervene? (during different stages of the life course and specific types of food/drink – like ultra processed food) (1) |  |  |  |
| 2. What is the role of **physical activity** in brain health and how can we intervene? (including amount and types of exercise) (2) |  |  |  |
| 3. What brain stimulating and lifelong **learning activities** are beneficial for brain health? (including amount, types, variety and suitability for different ages/abilities) (3) |  |  |  |
| 4. What **cultural activities** – like music, meditation, museum visits – are beneficial for brain health? (including amount and types) (4) |  |  |  |
| 5. What **social activities** are beneficial for brain health? (including types of community-group-based activities and intergenerational activities) (5) |  |  |  |
| 6. What is the role of **sleep** in brain health and how can we intervene? (including amount and sleep-related disorders) (6) |  |  |  |
| 7. What is the right **balance for lifestyle efforts** for an individual? (including magnitude of effect and strain of feeling obligated to improve) (7) |  |  |  |
| 8. What are **ways to reduce stress** over the life course – like a four day working week – and do these benefit brain health? (8) |  |  |  |
| 9. What **activities** that can be done **in care homes specifically** are beneficial for brain health? (9) |  |  |  |
| 10. What **mix** of physical, mental, cultural and social activities is most beneficial for brain health? (10) |  |  |  |
| 11. Are certain **types of social contact** – like groups versus one on one, online versus offline etc – more beneficial for brain health than others? (11) |  |  |  |

Q42 Interventions B. Medication and hospital treatments

|  | Take OUT of list (1) | Neutral (2) | Keep IN list (3) |
| --- | --- | --- | --- |
| 1. What **new treatments for preventing or stopping dementia** can be developed? (including acceptable side effects, optimal timing, applicability for subtypes of dementia) (1) |  |  |  |
| 2. What are ways to **improve hearing loss interventions** so that brain health is also benefitted? (2) |  |  |  |
| 3. Are there **existing drugs that can be repurposed** to help prevent dementia? (3) |  |  |  |
| 4. Is there a role for **gene therapy** in prevention of dementia? (4) |  |  |  |
| 5. What new interventions for the **symptoms of dementia** can be developed? (including distress interventions) (5) |  |  |  |
| 6. Is there a role for **vaccines** in prevention of dementia? (6) |  |  |  |

Q43 Interventions This is the last subtheme of this theme, so after this page you cannot change your answers. C. (Sub)population wide interventions

|  | Take OUT of list (1) | Neutral (2) | Keep IN list (3) |
| --- | --- | --- | --- |
| 1. What are interventions to benefit brain health that can be done at a **national, full population level** – like air pollution legislation, healthy food subsidies etc? (1) |  |  |  |
| 2. What are interventions to benefit brain health that can be done during **childhood, mid-life and old age populations**? (2) |  |  |  |
| 3. What interventions help **prevent falls and frailty** in older adults living with dementia? (3) |  |  |  |
| 4. What interventions are best for **different subpopulations** – like age groups, certain comorbidity like neurological disease? (including efficacy and cost) (4) |  |  |  |

Q49 Support Subtheme A-F	A. Community

|  | Take OUT of list (1) | Neutral (2) | Keep IN list (3) |
| --- | --- | --- | --- |
| 1. How can **community** (groups) aid brain health? (1) |  |  |  |
| 2. Could there be a role for local community **hubs**, for example in schools, as informal **information and support** point about brain health and dementia besides formal (NHS) routes? (2) |  |  |  |
| 3. How **accessible** are **community activities** for people (with dementia) in Scotland? (3) |  |  |  |
| 4. Should **local authorities support** community groups in their informal contribution to brain health; and if so, how? (4) |  |  |  |

Q50 Support B. Caregivers

|  | Take OUT of list (1) | Neutral (2) | Keep IN list (3) |
| --- | --- | --- | --- |
| 1. What support do caregivers **need** and do the current services meet their (different) needs? (1) |  |  |  |
| 2. How can **brain health of caregivers** be promoted? (2) |  |  |  |
| 3. When there is a **change in the caregiver status**, how can people be supported to maintain their employment and/or other **activities**? (3) |  |  |  |
| 4. How many **informal caregivers** are there in Scotland? (4) |  |  |  |

Q52 Support C. Technology

|  | Take OUT of list (1) | Neutral (2) | Keep IN list (3) |
| --- | --- | --- | --- |
| 1. What **technological applications** can help people living with dementia in their **day-to-day life**? (including quality of life, independence and compared to non-tech solutions) (1) |  |  |  |
| 2. How can **artificial intelligence** help improve (diagnostic and care) services around dementia and brain health? (including personalisation) (2) |  |  |  |

Q53 Support D. Independence

|  | Take OUT of list (1) | Neutral (2) | Keep IN list (3) |
| --- | --- | --- | --- |
| 1. What support can be given to let people live **independently and/or at home as long as possible**? (1) |  |  |  |
| 2. What factors are needed for people to remain **living at home responsibly** – like specific cognitive functions or caregiver abilities? (2) |  |  |  |

Q54 Support E. Social care

|  | Take OUT of list (1) | Neutral (2) | Keep IN list (3) |
| --- | --- | --- | --- |
| 1. How can we improve care for people living with **advanced dementia** so that their **quality of life** increases? (1) |  |  |  |
| 2. What should **integrated health and social care** look like for brain health? (2) |  |  |  |
| 3. What are specific **social care interventions** to support people in managing problems around brain health – like an intervention to minimise the negative impact losing driver’s license, talking therapies, etc? (12) |  |  |  |
| 4. How to optimise the **transition** of people living with dementia from their house into a care home or the hospital? (including timing, process and support) (13) |  |  |  |

Q51 Support This is the last subtheme of this theme, so after this page you cannot change your answers. F. The diagnosis journey

|  | Take OUT of list (1) | Neutral (2) | Keep IN list (3) |
| --- | --- | --- | --- |
| 1. In what ways can **post-diagnostic support** be optimised? (including setting where it is offered, timing and modification for certain groups like people with autism and care homes) (1) |  |  |  |
| 2. How can we make sure that people living with dementia are **prepared for future** challenges/decisions? (including what messages need to be given as support and by whom) (2) |  |  |  |
| 3. How can we support people who are **at risk of future poor brain health** (because of heredity or (young onset) dementia)? (3) |  |  |  |
| 4. How many people with possible dementia are **underdiagnosed**? (4) |  |  |  |
| 5. How can we best support those with **concerns** about brain health for themselves or a loved one (who might not agree)? (12) |  |  |  |
| 6. In what way can people be supported day-to-day to **make lifestyle changes** needed to improve brain health? (13) |  |  |  |

Q62 Awareness & Education Subtheme A-E	A. Campaigns

|  | Take OUT of list (1) | Neutral (2) | Keep IN list (3) |
| --- | --- | --- | --- |
| 1. How to **educate the general public about brain health** (including routes, subjects and how not to cause anxiety) and are **different methods** needed for **different groups** - like younger ages, people with no current brain health issues and people with additional communication needs? (1) |  |  |  |
| 2. What is the best way to **inform the general public** about all aspects of brain health **research**? (including brief summaries of the latest science, and how to get involved in research) (2) |  |  |  |
| 3. How can current **patient education** about brain health be improved? (3) |  |  |  |

Q63 Awareness & Education B. Stigma

|  | Take OUT of list (1) | Neutral (2) | Keep IN list (3) |
| --- | --- | --- | --- |
| 1. Would the **term ‘brain health’** and speaking about modifiable risk factors actually **increase stigma** for people with dementia that could not have been prevented? (1) |  |  |  |
| 2. What are **ways to reduce stigma** around dementia and normalise admitting you have declining brain health, and what is the role of media portrayals of dementia in this? (2) |  |  |  |
| 3. Does **decreased stigma** lead to **earlier detection** of dementia? (12) |  |  |  |

Q64 Awareness & Education C. Skills professionals

|  | Take OUT of list (1) | Neutral (2) | Keep IN list (3) |
| --- | --- | --- | --- |
| 1. How can we ensure that **all health professionals**, not just dementia specialists, provide **dementia friendly care**? (1) |  |  |  |
| 2. How to incorporate brain health and dementia better in **health and social care curricula**? (including postgraduate training for GPs and non-dementia specialists) (2) |  |  |  |
| 3. How can we optimise **listening and communication skills** of health professionals towards people with dementia? (12) |  |  |  |
| 4. How and to what level do we improve **standards of training** for care home and care at home **staff**? (13) |  |  |  |
| 5. How to make health professionals and the public more aware of the **variety of dementia subtypes** **and** (non-Alzheimer) **symptoms**? (14) |  |  |  |
| 6. How to improve training of professionals in **cognitive assessments and diagnosis**? (15) |  |  |  |
| 7. How do we give **assurance of training level** of health and social care professionals? (16) |  |  |  |

Q65 Awareness & Education D. Public view

|  | Take OUT of list (1) | Neutral (2) | Keep IN list (3) |
| --- | --- | --- | --- |
| 1. To what extent is the **importance** of brain health **recognised** through **Scotland**? (1) |  |  |  |
| 2. How can **working environments** be more **brain health friendly**? (2) |  |  |  |

Q67 Awareness & Education *This is the last subtheme of this theme, so after this page you cannot change your answers.* E. Skills caregivers

|  | Take OUT of list (1) | Neutral (2) | Keep IN list (3) |
| --- | --- | --- | --- |
| 1. What are (easily understandable) ways to **educate caregivers** to provide good dementia care? (1) |  |  |  |
| 2. What is the evidence base for **coping** with different **dementia symptoms** – like how to react when someone is confused about reality? (2) |  |  |  |
| 3. How do we **ensure a caregiver meets the standards** that can be expected **of caring** for a loved one? (3) |  |  |  |

Q68 Research Methods *Subtheme A-E*	A. Principle design of studies

|  | Take OUT of list (1) | Neutral (2) | Keep IN list (3) |
| --- | --- | --- | --- |
| 1. Can we use research to **follow people** from a young age to understand brain health in Scotland – like risk factors and interventions? (longitudinal cohort studies and trials) (1) |  |  |  |
| 2. What is the role of **big data** and how can routine data collection in Scotland be improved? (2) |  |  |  |
| 3. What **gene studies** can bring scientific advancements? (3) |  |  |  |
| 4. How can we set up more **interdisciplinary research collaborations**? (12) |  |  |  |
| 5. What is the role of **animal models** in brain health research? (13) |  |  |  |

Q69 Research Methods B. Measuring outcomes

|  | Take OUT of list (1) | Neutral (2) | Keep IN list (3) |
| --- | --- | --- | --- |
| 1. What are the right metrics to **measure brain health** in research? (1) |  |  |  |
| 2. How to **incorporate individual brain health priorities** into the clinical trial outcomes? (2) |  |  |  |

Q70 Research methods C. Patient and public involvement

|  | Take OUT of list (1) | Neutral (2) | Keep IN list (3) |
| --- | --- | --- | --- |
| 1. How can research (inclusion) be better **embedded into** the front line of **NHS**? (1) |  |  |  |
| 2. How can we motivate people to **donate their brain to science**? (2) |  |  |  |
| 3. What are **ways to increase** and improve patient and public **involvement** in brain health and dementia research? (12) |  |  |  |

Q71 Research methods D. Translation into practice

|  | Take OUT of list (1) | Neutral (2) | Keep IN list (3) |
| --- | --- | --- | --- |
| 1. How can we **improve implementation** of scientific advancements into (NHS) policy? (1) |  |  |  |
| 2. Could **evidence reviews** of current evidence help researchers to keep overview and prevent duplication? (2) |  |  |  |
| 3. How do we optimise the Scottish brain health and dementia research space to **gain worldwide visibility**? (12) |  |  |  |

Q72 Research Methods *This is the last subtheme of this theme, so after this page you cannot change your answers.* E. Participant communication

|  | Take OUT of list (1) | Neutral (2) | Keep IN list (3) |
| --- | --- | --- | --- |
| 1. How can we ethically gain **informed consent** from people living with dementia? (1) |  |  |  |
| 2. How can we **support** people participating in dementia and brain health studies, **before, during and after the study**? (2) |  |  |  |

Q73 System & Logistics *Subtheme A-C*	A. Care pathways

|  | Take OUT of list (1) | Neutral (2) | Keep IN list (3) |
| --- | --- | --- | --- |
| 1. How can we optimise the **brain health and dementia care path in Scotland**? (including personalisation, ensuring follow-up, designing new “memory clinics”, and cross-disciplinary links) (1) |  |  |  |
| 2. What can we learn from **other countries**’ dementia and brain health care systems? (including assisted dying law) (2) |  |  |  |
| 3. What role can **clinicians outside traditional medical hospital services** – like allied health professionals and primary care music therapists – play in promoting brain health through the life course? (3) |  |  |  |
| 4. How do we support **care homes** to provide **more complex care**, considering high pressure on NHS hospital wards? (12) |  |  |  |
| 5. What are the benefits and limitations of doing **annual care reviews** for people living with dementia? (13) |  |  |  |
| 6. How are **paid and unpaid** dementia care **services interacting**? (14) |  |  |  |

Q75 System & Logistics B. Buildings

|  | Take OUT of list (1) | Neutral (2) | Keep IN list (3) |
| --- | --- | --- | --- |
| 1. How can hospitals, care homes, and care at home services be **supported** to get **infrastructure** to provide quality of life increasing **activities** – like going outdoors, music etc? (1) |  |  |  |
| 2. Are certain **room designs** – like amount of natural light – beneficial for quality of life of people living with dementia? (2) |  |  |  |
| 3. How can **public spaces** be improved to be more **dementia friendly**? (12) |  |  |  |

Q77 System & Logistics *This is the last subtheme of this theme, so after this page you cannot change your answers.* C. Financing

|  | Take OUT of list (1) | Neutral (2) | Keep IN list (3) |
| --- | --- | --- | --- |
| 1. How can we **financially prepare** our health care system for the **future**, considering the ageing population? (1) |  |  |  |
| 2. Are there better ways to **finance our care workforce**? (including dementia link nurses) (2) |  |  |  |
| 3. What are the benefits and limitations of various **models of care home funding**? (14) |  |  |  |

Q78 Equity *Subtheme A-B*	A. Evaluation of inequalities

|  | Take OUT of list (1) | Neutral (2) | Keep IN list (3) |
| --- | --- | --- | --- |
| 1. Are there **socio-economic inequalities** in brain health, dementia prevalence and quality of life for people living with dementia, and how do we intervene? (1) |  |  |  |
| 2. What are differences in brain health and dementia care between **urban and rural areas of Scotland**? (2) |  |  |  |

Q80 Equity *This is the last subtheme of this theme, so after this page you cannot change your answers.* B. Inclusion in care and research

|  | Take OUT of list (1) | Neutral (2) | Keep IN list (3) |
| --- | --- | --- | --- |
| 1. How can we ensure everyone has **equal access** to brain health **services** and dementia treatment, diagnostic tools and caregiver support? (including neurodivergent people) (1) |  |  |  |
| 2. How can we better **include underrepresented groups** in brain health **research** and **minimise the current disparities in evidence base**? (including – but not limited to – knowledge gaps for female gender, non-white ethnicity and frail people) (2) |  |  |  |
| 3. Is there **decreased accessibility to health care** for people living with dementia, **or the other way around** people with other conditions having less access to dementia care? (3) |  |  |  |
| 4. How can we ensure interventions don’t **exacerbate existing inequalities**? (4) |  |  |  |

### Survey C (including full short list)

Q1 Thank you for participating in this third survey. We highly value your opinion. Our aim is to find out which are the most important questions for future brain health research. In this project we are interested in the views of adults living in Scotland. We want to hear the opinions of people like you, who have an interest in dementia and brain health. This will make sure that research addresses topics that matter to the people affected.

Q2 In this survey we will ask you to rank brain health research questions that were graded in the previous survey. The long list resulting from a first questionnaire that collected research topics was shortened by asking for each question if it should be out, neutral or in the priority list. This brings us yet again a step closer to establishing the Scottish top 10 research priorities for brain health. This top 10 will be given to research funding bodies among others.

We will also collect demographic details. This is to find out more about who has completed the survey. **It will not be possible to identify any individual respondent from this survey.** If at any point you feel like you do not want to take part anymore, you are free to stop the survey. Your participation is entirely voluntary. Also, it is possible to take a break and finish the survey later.

Q27 Please find full study information and the privacy notice below. You can also find the terms that make up your consent, if you agree to participate. Ticking the boxes below suffices as consent, you don't need to email anything to us.

All documents are the same as the previous survey. The links will open in new tabs.

1. Participant information sheet surveys 310325

2. Privacy notice surveys 310325

3. Consent form surveys 310325

o	I declare that I am 18 years or older (4)

Q28 and

o	I have read the terms as described in the documents above and agree to participate in this study (1)

Q3 Thank you! Your consent has been registered. As filling in online surveys may be challenging, feel free to ask someone to assist you. If you would rather give your views in another way, please email. Then we can explore what fits best, such as a phone appointment or printed survey by post. Thanks again and let's get started!

Q18 First, we need to collect a little information about you again. This way, we can check how well the people of Scotland are represented in our survey and if different groups prioritise different topics

Q19 What best describes your role?

o	Person living with dementia (1)

o	Person living with mild cognitive impairment (2)

o	Caregiver (3)

o	Former caregiver (4)

o	Researcher (5)

o	Clinician (doctor, nurse, paramedic, professional caretaker, allied health professional, etc) (6)

o	Third sector (policy, charity, etc) (7)

o	Scottish citizen with interest in brain health (for example because you have family members with dementia) (8)

o	Other, that is: (9) __________________________________________________

Q20 In what type of location are you currently living?

o	Large city (1)

o	Suburb near a large city (2)

o	Small city or town (3)

o	Rural area (4)

o	Other (5)

o	Prefer not to say (6)

Q21 What gender do you self-identify with?

o	Male (1)

o	Female (2)

o	Non-binary / third gender (3)

o	Prefer not to say (4)

Q25 Which ethnicity do you identify with?

o	White (1)

o	Mixed or multiple (2)

o	Asian, Asian Scottish or Asian British (3)

o	Caribbean or Black (4)

o	Other (5)

o	Prefer not to say (6)

Q22 What age group do you fall in? *If you are under 18, unfortunately you cannot participate in this survey and we kindly ask you to close this tab.*

o	18-29 years old (1)

o	30-39 years old (2)

o	40-49 years old (3)

o	50-59 years old (4)

o	60-69 years old (5)

o	70-79 years old (6)

o	80-89 years old (7)

o	90-99 years old (8)

o	>100 years old (9)

o	Prefer not to say (10)

Q32 Did you participate in the previous survey, which collected research topics? If not, you will be redirected to a page with an explanation about the concept of brain health as we introduced it in the first survey.

o	Yes (1)

o	Yes, but i would like to see the explanation again (2)

o	No (4)

Q34 **Quick background** There are many definitions of brain health. All definitions agree that brain health is more than just absence of diseases like dementia. Brain health means keeping your brain function at the highest level possible, for as long as possible. While many brain diseases are seen in older adults, brain health is important throughout your whole life. Recent research has identified factors that can cause poor brain health. These include poor sleep, air pollution and limited access to education. It is estimated that up to 40% dementia diagnoses worldwide could be prevented by attending to these factors. There may be other factors that are bad for brain health, or can protect brain health. Research could help identify these factors. Research could also describe how to reduce the problems from factors such as poor sleep.

You may have responded to the invitation to participate as you are a person living with dementia, or a care partner of someone with dementia. This is also included in this study. However, our brain health focus means we do not have to limit our thoughts to dementia but cover the whole life course spectrum of (maintaining and declining) brain health.

Q7 **Explaining the current priority list**

We are currently in the ranking phase of our study. Eventually we want to end up with a national top 10 list of research priorities for brain health. We build consensus through multiple rounds of ranking.

This round we will do the ranking by establishing a preferred order of the remaining items. This will result in a shorter list of priorities and if another round is needed, in a next survey you will be asked to do the same.

After collecting all 2676 research topics people submitted in the previous survey, we collated this in a long list. This process resulted in a list of 142 questions in 8 themes and 34 subthemes. The first grading survey (out, neutral, in) shortened the list to 50 questions in order of priority so far

Q44 **Set up of this third survey**

We kindly ask you to go through the 50 questions that remained. First you can select which 10 research questions you think are most important.

After, on a new page you are asked to put them in the order of your priorities. To sum up, you create your personal top 10

Choose questions Could you select 10 questions out of this list of 50 that you think are most important?

The questions are numbered based on results of the previous survey. Some questions had exactly the same scores, so they will have a letter after the number.

▢	1. What are ways to diagnose dementia earlier than in current practice? (including identifying key early symptoms and biomarkers) (1)

▢	2. What new treatments for preventing or stopping dementia can be developed? (including acceptable side effects, optimal timing, applicability for subtypes of dementia) (2)

▢	3A. How much can people – especially those with high genetic or other risk for dementia – reduce their risk? (3)

▢	3B. How can we financially prepare our health care system for the future, considering the ageing population? (4)

▢	5. How can we optimise the brain health and dementia care path in Scotland? (including personalisation, ensuring follow-up, designing new “memory clinics”, and cross-disciplinary links) (5)

▢	6. What support can be given to let people live independently and/or at home as long as possible? (6)

▢	7. How can we improve care for people living with advanced dementia so that their quality of life increases? (7)

▢	8. What brain stimulating and lifelong learning activities are beneficial for brain health? (including amount, types, variety and suitability for different ages/abilities) (8)

▢	9. What support do caregivers need and do the current services meet their (different) needs? (9)

▢	10. What are (easily understandable) ways to educate caregivers to provide good dementia care? (10)

▢	11. What is the role of sleep in brain health and how can we intervene? (including amount and sleep-related disorders) (11)

▢	12A. Which is the most important factor contributing to brain health? (12)

▢	12B. What factors are needed for people to remain living at home responsibly – like specific cognitive functions or caregiver abilities? (13)

▢	14A. Are there existing drugs that can be repurposed to help prevent dementia? (14)

▢	14B. How and to what level do we improve standards of training for care home and care at home staff? (15)

▢	16A. How can we ensure that all health professionals, not just dementia specialists, provide dementia friendly care? (16)

▢	16B. How to incorporate brain health and dementia better in health and social care curricula? (including postgraduate training for GPs and non-dementia specialists) (17)

▢	18A. What is the role of stress and anxiety in brain health? (including different sources, amounts and length of exposure) (18)

▢	18B. How can brain scans best be used in brain health care? (including predicting dementia risk and following brain changes over time) (19)

▢	20A. What is the role of diet in brain health and how can we intervene? (during different stages of the life course and specific types of food/drink – like ultra processed food) (20)

▢	20B. How can we support people who are at risk of future poor brain health (because of heredity or (young onset) dementia)? (21)

▢	22. What is the role of physical activity in brain health and how can we intervene? (including amount and types of exercise) (22)

▢	23. How to improve training of professionals in cognitive assessments and diagnosis? (23)

▢	24A. What is the role of long-term health conditions – like heart disease, diabetes, auto-immune disease etc – in brain health? (24)

▢	24B. What new interventions for the symptoms of dementia can be developed? (including distress interventions) (25)

▢	24C. How do we support care homes to provide more complex care, considering high pressure on NHS hospital wards? (26)

▢	24D. How can we ensure everyone has equal access to brain health services and dementia treatment, diagnostic tools and caregiver support? (including neurodivergent people) (27)

▢	28. What is the role of social connectedness in brain health? (including social isolation, owning a pet, receiving kindness) (28)

▢	29. Are there better ways to finance our care workforce? (including dementia link nurses) (29)

▢	30. Why do some people – especially those with high genetic or other risk on paper – keep good brain health in reality? (including brain resilience) (30)

▢	31. What is the role of working life factors in brain health? (including type of occupation and retirement age) (31)

▢	32A. What is the evidence base for coping with different dementia symptoms – like how to react when someone is confused about reality? (32)

▢	32B. How can we improve implementation of scientific advancements into (NHS) policy? (33)

▢	34A. What is the role of synthetics in consumer products - like food additives, microplastics and pesticides - in brain health? (34)

▢	34B. What is the role of commonly prescribed medication use in brain health? (including proton pump inhibitors, antidepressants, statins etc) (35)

▢	36. What is the role of differing forms and severities of traumatic brain injury in brain health? (36)

▢	37. What are interventions to benefit brain health that can be done during childhood, mid-life and old age populations? (37)

▢	38A. Does an early diagnosis of dementia actually benefit the person and their family, considering currently available treatments? (38)

▢	38B. How can we make sure that people living with dementia are prepared for future challenges/decisions? (including what messages need to be given as support and by whom) (39)

▢	40. What can we learn from other countries’ dementia and brain health care systems? (including assisted dying law) (40)

▢	41A. Considering accuracy and acceptability, what is the best way to measure cognitive function in the Scottish context – like pencil and paper tests, questionnaires or computers? (41)

▢	41B. What social activities are beneficial for brain health? (including types of community-group-based activities and intergenerational activities) (42)

▢	41C. What mix of physical, mental, cultural and social activities is most beneficial for brain health? (43)

▢	44A. In what way can people be supported day-to-day to make lifestyle changes needed to improve brain health? (44)

▢	44B. What gene studies can bring scientific advancements? (45)

▢	46A. How can we fully understand the biology of dementia (subtypes)? (46)

▢	46B. What should integrated health and social care look like for brain health? (47)

▢	46C. Can we use research to follow people from a young age to understand brain health in Scotland – like risk factors and interventions? (longitudinal cohort studies and trials) (48)

▢	49A. How can we optimise listening and communication skills of health professionals towards people with dementia? (49)

▢	49B. What are ways to increase and improve patient and public involvement in brain health and dementia research? (50)

Could you now put these 10 into the order that you think the priority list should have? By holding a question with your mouse/finger you can drag and drop it at another place/rank in the list.

When filling out the survey on your smartphone, mind that scrolling down you don't accidentally move the priorities. There is a thin slider to scroll down on the right side of your screen.

Q23 That was it!

If there is any feedback (on either this survey or the prioritisation research in general), feel free to write it here. Or let us know if there was a question you did not understand.

________________________________________________________________

Q24 **Looking forward**

The responses will be collected, and after combining all answers the list can be shortened further. You can be invited to partake in the next round. This depends on how much shorter the list is after this round of ranking. Study end results will be shared through the same platforms used for finding participants

### Survey D

Q1 Thank you for participating in this fourth and last survey of our study. We highly value your opinion. Our aim is to find out which are the most important questions for future brain health research. This will make sure that research addresses topics that matter to the people affected. In this project we have been interested in the views of adults living in Scotland. However, this last survey is an exception: we are interested in the views of dementia research experts.

Q2 In this survey we will ask you to appraise the impact per topic for two interim research priority lists: one for dementia and one for brain health. This appraisal will decide the order of the final rankings. The top tens will be distributed to research funding bodies among others to guide the Scottish research agenda.

We will also collect some demographic details. This is to find out more about who has completed the survey. **It will not be possible to identify any individual respondent from this survey.** If at any point you feel like you do not want to take part anymore, you are free to stop the survey. Your participation is entirely voluntary. Also, it is possible to take a break and finish the survey later.

Q27 Please find full study information and the privacy notice below. You can also find the terms that make up your consent, if you agree to participate. Ticking the boxes below suffices as consent, you don't need to email anything to us.

All documents are the same as previous surveys, if you have already participated before. The links will open in new tabs.

1. Participant information sheet surveys 310325

2. Privacy notice surveys 310325

3. Consent form surveys 310325

o	I declare that I am 18 years or older (4)

Q28 and

o	I have read the terms as described in the documents above and agree to participate in this study (1)

Q3 Thank you! Your consent has been registered.

Q18 First, we need to collect a little information about you. This way, we can check how well the dementia research experts of Scotland are represented in our survey.

Q19 What university are you affiliated with?

o	University of Glasgow (1)

o	University of Edinburgh (2)

o	University of Aberdeen (3)

o	University of Dundee (4)

o	University of Strathclyde (5)

o	Glasgow Caledonian University (6)

o	University of the West of Scotland (7)

o	University of St Andrews (8)

o	Other university, that is: (9) ______________________________________________

o	Not a university, that is: (10) ______________________________________________

Q20 What type of research field are you in?

o	Clinical research (1)

o	Data research (2)

o	Translational science (3)

o	Other (4) __________________________________________________

Q21 What gender do you identify with?

o	Male (1)

o	Female (2)

o	Non-binary / third gender (3)

o	Prefer not to say (4)

Q22 How many years have you been working in dementia research?

o	0-5 years (1)

o	5-10 years (2)

o	10-15 years (3)

o	15-20 years (4)

o	20-25 years (5)

o	25-30 years (6)

o	>30 years (7)

Q26 Thank you. We now go on to the second part of the survey

Q32 Did you participate in the previous surveys, which collected research topics and ranked a long, collated list? *If not, you will be redirected to a page with an explanation about the concept of brain health.*

o	Yes (1)

o	Yes, but i would like to see the explanation again (2)

o	No (4)

Q34 **Quick background** There are many definitions of brain health. All definitions agree that brain health is more than just absence of diseases like dementia. Brain health means keeping your brain function at the highest level possible, for as long as possible. While many brain diseases are seen in older adults, brain health is important throughout your whole life. Recent research has identified factors that can cause poor brain health. These include poor sleep, air pollution and limited access to education. It is estimated that up to 40% dementia diagnoses worldwide could be prevented by attending to these factors. There may be other factors that are bad for brain health, or can protect brain health. Research could help identify these factors. Research could also describe how to reduce the problems from factors such as poor sleep.

You may have responded to the invitation to participate as you are a person living with dementia, or a care partner of someone with dementia. This is also included in this study. However, our brain health focus means we do not have to limit our thoughts to dementia but cover the whole life course spectrum of (maintaining and declining) brain health.

Q7 **Explaining the current priority lists**

We are currently in the ranking phase of our study. We started with 2676 research topics that >700 people submitted, and have come a long way: collating it into 142 research questions, and subsequently shortening the list to 50 and now 20 questions, building consensus through Delphi style ranking surveys. The interest-holders that have contributed are people living with dementia/mild cognitive impairment, caregivers, clinicians, researchers, third sector parties, and Scottish citizens with an interest in brain health.

If you were not yet participating but are interested in these previous steps, feel free to reach out for more information.

This will be the last survey round of this study. Based on one longer list, we chose to split up the priorities into dementia-oriented and brain-health-oriented research questions. We hope the split will help bring the importance of both overlapping topics forward to both the public as research funding bodies, because there are just too many important research questions to combine them into one priority list.

Q44 **Set up of this last survey**

We kindly ask you to appraise five criteria items for each question. The criteria are derived from a WHO's priority setting exercise (Shah et al., 2020), and are as followed:

- Feasibility of study: i.e. potential for success (“To the best of your knowledge and experience, would you say that the proposed research would likely be successful in reaching the proposed endpoint within the next decade?”)
- Reduce burden: i.e. magnitude effect (“If successful, would you say that this research has a potential to markedly reduce the burden of dementia on patients, caregivers and the society as a whole?”)
- Novelty: i.e. potential for conceptual breakthrough (“If successful, would you say that this research is likely to result in a paradigm shift [‘game changer’] that would fundamentally change our thinking or approach to the challenge of dementia?”)
- Implementation: i.e. potential for translation (“If successful, would you say that the proposed research would likely lead to practical application, implementation of new knowledge and/or be deliverable at scale?”)
- Equity: i.e. fairness and inclusiveness (“If successful, would you say that the proposed research outcome is likely to benefi t people living with or at risk of dementia, their carers, and societies as a whole in an equitable manner? Please consider countries, cultures, ethnicities, socio-economic status, gender, age, and any other relevant factors”)

The options will be a 5-point Likert scale (ranging from 'absolutely not' to 'absolutely yes'). The order of the top tens as showed in this survey is based on how high they scored on average in previous ranking surveys

Q68 Could you now score these 10 **brain health research priorities** on the impact criteria?

For reference, the accompanying questions per criterium are shown again:

- Feasibility of study: “To the best of your knowledge and experience, would you say that the proposed research would likely be successful in reaching the proposed endpoint within the next decade?”
- Reduce burden: “If successful, would you say that this research has a potential to markedly reduce the burden of dementia on patients, caregivers and the society as a whole?”
- Novelty: “If successful, would you say that this research is likely to result in a paradigm shift [‘game changer’] that would fundamentally change our thinking or approach to the challenge of dementia?”
- Implementation: “If successful, would you say that the proposed research would likely lead to practical application, implementation of new knowledge and/or be deliverable at scale?”
- Equity: “If successful, would you say that the proposed research outcome is likely to benefit people living with or at risk of dementia, their carers, and societies as a whole in an equitable manner? Please consider countries, cultures, ethnicities, socio-economic status, gender, age, and any other relevant factors”

Q 1. **How much** can people - especially those with high genetic or other risk for dementia - **reduce** their **risk**?

|  | Absolutely not (1) | Likely not (2) | Not sure/I do not know (3) | Likely yes (4) | Absolutely yes (5) |
| --- | --- | --- | --- | --- | --- |
| Feasibility of study (1) |  |  |  |  |  |
| Reduce burden (2) |  |  |  |  |  |
| Novelty (3) |  |  |  |  |  |
| Implementation (4) |  |  |  |  |  |
| Equity (5) |  |  |  |  |  |

Q67 2. What brain stimulating and lifelong **learning activities** are beneficial for brain health? (including amount, types, variety and suitability for different ages/abilities)

|  | Absolutely not (1) | Likely not (2) | Not sure/I do not know (3) | Likely yes (4) | Absolutely yes (5) |
| --- | --- | --- | --- | --- | --- |
| Feasibility of study (1) |  |  |  |  |  |
| Reduce burden (2) |  |  |  |  |  |
| Novelty (3) |  |  |  |  |  |
| Implementation (4) |  |  |  |  |  |
| Equity (5) |  |  |  |  |  |

Q69 3. How can we **financially prepare** our health care system **for the future**, considering the ageing population?

|  | Absolutely not (1) | Likely not (2) | Not sure/I do not know (3) | Likely yes (4) | Absolutely yes (5) |
| --- | --- | --- | --- | --- | --- |
| Feasibility of study (1) |  |  |  |  |  |
| Reduce burden (2) |  |  |  |  |  |
| Novelty (3) |  |  |  |  |  |
| Implementation (4) |  |  |  |  |  |
| Equity (5) |  |  |  |  |  |

Q70 4. Which is the **most important factor** contributing to brain health?

|  | Absolutely not (1) | Likely not (2) | Not sure/I do not know (3) | Likely yes (4) | Absolutely yes (5) |
| --- | --- | --- | --- | --- | --- |
| Feasibility of study (1) |  |  |  |  |  |
| Reduce burden (2) |  |  |  |  |  |
| Novelty (3) |  |  |  |  |  |
| Implementation (4) |  |  |  |  |  |
| Equity (5) |  |  |  |  |  |

Q71 5. What is the role of **diet** in brain health and how can we intervene? (during different stages of the life course and specific types of food/drink - like ultra processed food)

|  | Absolutely not (1) | Likely not (2) | Not sure/I do not know (3) | Likely yes (4) | Absolutely yes (5) |
| --- | --- | --- | --- | --- | --- |
| Feasibility of study (1) |  |  |  |  |  |
| Reduce burden (2) |  |  |  |  |  |
| Novelty (3) |  |  |  |  |  |
| Implementation (4) |  |  |  |  |  |
| Equity (5) |  |  |  |  |  |

Q72 6. What is the role of **sleep** in brain health and how can we intervene? (including amount and sleep-related disorders)

|  | Absolutely not (1) | Likely not (2) | Not sure/I do not know (3) | Likely yes (4) | Absolutely yes (5) |
| --- | --- | --- | --- | --- | --- |
| Feasibility of study (1) |  |  |  |  |  |
| Reduce burden (2) |  |  |  |  |  |
| Novelty (3) |  |  |  |  |  |
| Implementation (4) |  |  |  |  |  |
| Equity (5) |  |  |  |  |  |

Q73 7. What is the role of **physical activity** in brain health and how can we intervene? (including amount and types of exercise)

|  | Absolutely not (1) | Likely not (2) | Not sure/I do not know (3) | Likely yes (4) | Absolutely yes (5) |
| --- | --- | --- | --- | --- | --- |
| Feasibility of study (1) |  |  |  |  |  |
| Reduce burden (2) |  |  |  |  |  |
| Novelty (3) |  |  |  |  |  |
| Implementation (4) |  |  |  |  |  |
| Equity (5) |  |  |  |  |  |

Q74 8. What is the role of **stress and anxiety** in brain health? (including different sources, amounts and length of exposure)

|  | Absolutely not (1) | Likely not (2) | Not sure/I do not know (3) | Likely yes (4) | Absolutely yes (5) |
| --- | --- | --- | --- | --- | --- |
| Feasibility of study (1) |  |  |  |  |  |
| Reduce burden (2) |  |  |  |  |  |
| Novelty (3) |  |  |  |  |  |
| Implementation (4) |  |  |  |  |  |
| Equity (5) |  |  |  |  |  |

Q75 9. What are **interventions** to benefit brain health that can be done during **childhood, mid-life and old age** populations?

|  | Absolutely not (1) | Likely not (2) | Not sure/I do not know (3) | Likely yes (4) | Absolutely yes (5) |
| --- | --- | --- | --- | --- | --- |
| Feasibility of study (1) |  |  |  |  |  |
| Reduce burden (2) |  |  |  |  |  |
| Novelty (3) |  |  |  |  |  |
| Implementation (4) |  |  |  |  |  |
| Equity (5) |  |  |  |  |  |

Q76 10. Why do **some people** - especially those with high genetic or other risk on paper - **keep good brain health** in reality? (including brain resilience)

|  | Absolutely not (1) | Likely not (2) | Not sure/I do not know (3) | Likely yes (4) | Absolutely yes (5) |
| --- | --- | --- | --- | --- | --- |
| Feasibility of study (1) |  |  |  |  |  |
| Reduce burden (2) |  |  |  |  |  |
| Novelty (3) |  |  |  |  |  |
| Implementation (4) |  |  |  |  |  |
| Equity (5) |  |  |  |  |  |

Q77 Could you now score these 10 **dementia research priorities** on the impact criteria?

For reference, the accompanying questions per criterium are shown again:

- Feasibility of study: “To the best of your knowledge and experience, would you say that the proposed research would likely be successful in reaching the proposed endpoint within the next decade?”
- Reduce burden: “If successful, would you say that this research has a potential to markedly reduce the burden of dementia on patients, caregivers and the society as a whole?”
- Novelty: “If successful, would you say that this research is likely to result in a paradigm shift [‘game changer’] that would fundamentally change our thinking or approach to the challenge of dementia?”
- Implementation: “If successful, would you say that the proposed research would likely lead to practical application, implementation of new knowledge and/or be deliverable at scale?”
- Equity: “If successful, would you say that the proposed research outcome is likely to benefit people living with or at risk of dementia, their carers, and societies as a whole in an equitable manner? Please consider countries, cultures, ethnicities, socio-economic status, gender, age, and any other relevant factors”

Q78 1. What are ways to **diagnose dementia earlier** than in current practice? (including identifying key early symptoms and biomarkers)

|  | Absolutely not (1) | Likely not (2) | Not sure/I do not know (3) | Likely yes (4) | Absolutely yes (5) |
| --- | --- | --- | --- | --- | --- |
| Feasibility of study (1) |  |  |  |  |  |
| Reduce burden (2) |  |  |  |  |  |
| Novelty (3) |  |  |  |  |  |
| Implementation (4) |  |  |  |  |  |
| Equity (5) |  |  |  |  |  |

Q79 2. What new **treatments for preventing or stopping dementia** can be developed? (including acceptable side effects, optimal timing, applicability for subtypes of dementia)

|  | Absolutely not (1) | Likely not (2) | Not sure/I do not know (3) | Likely yes (4) | Absolutely yes (5) |
| --- | --- | --- | --- | --- | --- |
| Feasibility of study (1) |  |  |  |  |  |
| Reduce burden (2) |  |  |  |  |  |
| Novelty (3) |  |  |  |  |  |
| Implementation (4) |  |  |  |  |  |
| Equity (5) |  |  |  |  |  |

Q80 3. How can we **optimise** the brain health and dementia **care path** in Scotland? (including personalisation, ensuring follow-up, designing new 'memory clinics', and cross-disciplinary links)

|  | Absolutely not (1) | Likely not (2) | Not sure/I do not know (3) | Likely yes (4) | Absolutely yes (5) |
| --- | --- | --- | --- | --- | --- |
| Feasibility of study (1) |  |  |  |  |  |
| Reduce burden (2) |  |  |  |  |  |
| Novelty (3) |  |  |  |  |  |
| Implementation (4) |  |  |  |  |  |
| Equity (5) |  |  |  |  |  |

Q81 4. Are there **existing drugs** that can be **repurposed** to help prevent dementia?

|  | Absolutely not (1) | Likely not (2) | Not sure/I do not know (3) | Likely yes (4) | Absolutely yes (5) |
| --- | --- | --- | --- | --- | --- |
| Feasibility of study (1) |  |  |  |  |  |
| Reduce burden (2) |  |  |  |  |  |
| Novelty (3) |  |  |  |  |  |
| Implementation (4) |  |  |  |  |  |
| Equity (5) |  |  |  |  |  |

Q82 5. What **support** can be given to let people **live independently** and/or at home as long as possible?

|  | Absolutely not (1) | Likely not (2) | Not sure/I do not know (3) | Likely yes (4) | Absolutely yes (5) |
| --- | --- | --- | --- | --- | --- |
| Feasibility of study (1) |  |  |  |  |  |
| Reduce burden (2) |  |  |  |  |  |
| Novelty (3) |  |  |  |  |  |
| Implementation (4) |  |  |  |  |  |
| Equity (5) |  |  |  |  |  |

Q83 6. How can we improve **care** for people living with **advanced dementia** so that their **quality of life** increases?

|  | Absolutely not (1) | Likely not (2) | Not sure/I do not know (3) | Likely yes (4) | Absolutely yes (5) |
| --- | --- | --- | --- | --- | --- |
| Feasibility of study (1) |  |  |  |  |  |
| Reduce burden (2) |  |  |  |  |  |
| Novelty (3) |  |  |  |  |  |
| Implementation (4) |  |  |  |  |  |
| Equity (5) |  |  |  |  |  |

Q84 7. What **support** do **caregivers need** and do the current services meet their (different) needs?

|  | Absolutely not (1) | Likely not (2) | Not sure/I do not know (3) | Likely yes (4) | Absolutely yes (5) |
| --- | --- | --- | --- | --- | --- |
| Feasibility of study (1) |  |  |  |  |  |
| Reduce burden (2) |  |  |  |  |  |
| Novelty (3) |  |  |  |  |  |
| Implementation (4) |  |  |  |  |  |
| Equity (5) |  |  |  |  |  |

Q85 8. How can we ensure that **all health professionals**, not just dementia specialists, provide **dementia friendly care**?

|  | Absolutely not (1) | Likely not (2) | Not sure/I do not know (3) | Likely yes (4) | Absolutely yes (5) |
| --- | --- | --- | --- | --- | --- |
| Feasibility of study (1) |  |  |  |  |  |
| Reduce burden (2) |  |  |  |  |  |
| Novelty (3) |  |  |  |  |  |
| Implementation (4) |  |  |  |  |  |
| Equity (5) |  |  |  |  |  |

Q86 9. How can **brain scans** best be used in brain health care? (including predicting dementia risk and following brain changes over time)

|  | Absolutely not (1) | Likely not (2) | Not sure/I do not know (3) | Likely yes (4) | Absolutely yes (5) |
| --- | --- | --- | --- | --- | --- |
| Feasibility of study (1) |  |  |  |  |  |
| Reduce burden (2) |  |  |  |  |  |
| Novelty (3) |  |  |  |  |  |
| Implementation (4) |  |  |  |  |  |
| Equity (5) |  |  |  |  |  |

Q87 10. What can we **learn from other countries**' dementia and brain health care systems? (including assisted dying law)

|  | Absolutely not (1) | Likely not (2) | Not sure/I do not know (3) | Likely yes (4) | Absolutely yes (5) |
| --- | --- | --- | --- | --- | --- |
| Feasibility of study (1) |  |  |  |  |  |
| Reduce burden (2) |  |  |  |  |  |
| Novelty (3) |  |  |  |  |  |
| Implementation (4) |  |  |  |  |  |
| Equity (5) |  |  |  |  |  |

Q23 That was it! We would be keen to hear any feedback on the questions that did (not) end up in the top tens or reasoning you would like to share.

If you have feedback on either this survey or research priority setting exercises in general, feel free to write it here as well.

______________________________________________________

## **Appendix 7: demographics survey D**

| Question | Answer options | Participants (total *n*=17) |
| --- | --- | --- |
| University affiliations | University of Glasgow | 5 (29·4%) |
|  | University of Edinburgh | 2 (11·8%) |
|  | University of Aberdeen | 2 (11·8%) |
|  | Other university* | 3 (17·6%) |
|  | Not a university** | 5 (29·4%) |
| Type of research field | Clinical research | 11 (64·7%) |
|  | Data research | 2 (11·8%) |
|  | Translational science | 3 (17·7%) |
|  | Other‡ | 1 (5·9%) |
| Gender | Male | 7 (41·2%) |
|  | Female | 9 (52·9%) |
|  | Non-binary / third gender | 0 (-) |
|  | Prefer not to say | 1 (5·9%) |
| Years experience dementia research | 0-5 years | 2 (11·8%) |
|  | 5-10 years | 1 (5·9%) |
|  | 10-15 years | 2 (11·8%) |
|  | 15-20 years | 3 (17·7%) |
|  | 20-25 years | 2 (11·8%) |
|  | 25-30 years | 1 (5·9%) |
|  | >30 years | 6 (35·3%) |

**Demographic details of survey D respondents** All categories are self-identified; * University of St Andrews, Heriot-Watt University, Edinburgh Napier University; ** National Health Service (NHS); ‡ reported as ‘cross-discipline’.

## **Appendix 8: interim list themes**

| Themes | Subthemes (*t*) | Questions (*q*) |
| --- | --- | --- |
| Pathology | 7 | 36 |
| Diagnosis | 3 | 12 |
| Interventions | 3 | 21 |
| Support | 6 | 22 |
| Awareness & Education | 5 | 18 |
| Research methods | 5 | 15 |
| System & Logistics | 3 | 12 |
| Equity | 2 | 6 |

**Amount of questions and subthemes per theme in interim list** These were presented to participants in survey B. Scoring of questions was done per theme.

**Appendix 9: Delphi 1 analysis age groups**

**Stratification of survey B outcome for age group.** Comparing overall ranking after Delphi 1 with ranking of sub groups (age 18-39, age 40-59, age 60-79, and age >80 years old. Top 50 in the overall ranking was taken to Delphi 2 round. Numbers are all ranking in overall group, so that it can be compared.

38 Does an early diagnosis of dementia actually benefit the person and their family, considering currently available treatments?

86 How can we better include underrepresented groups in brain health research and minimise the current disparities in evidence base? (including – but not limited to – knowledge gaps for female gender, non-white ethnicity and frail people)

64 What is the role of female hormones and its fluctuations through life in brain health? (including menopause, menstrual cycle and hormone replacement therapy (including for transpeople))

78 What is considered normal ageing and what is the threshold for poor brain health?

89 Are there socio-economic inequalities in brain health, dementia prevalence and quality of life for people living with dementia, and how do we intervene?

81 What is the role of (childhood) trauma and post-traumatic stress disorder (PTSD) in brain health?

62 How to educate the general public about brain health (including routes, subjects and how not to cause anxiety) and are different methods needed for different groups - like younger ages, people with no current brain health issues and people with additional communication needs?

52 What cultural activities – like music, meditation, museum visits – are beneficial for brain health? (including amount and types)

56 What activities that can be done in care homes specifically are beneficial for brain health?

75 How do we ensure a caregiver meets the standards that can be expected of caring for a loved one?

85 How can we support people participating in dementia and brain health studies, before, during and after the study?

## **References**

1.	Tan A, Nagraj SK, Nasser M, Sharma T, Kuchenmuller T. What do we know about evidence-informed priority setting processes to set population-level health-research agendas: an overview of reviews. *Bull Natl Res Cent.* 2022;**46**(1):6.

2.	National Records of Scotland. Scotland's Census 2022 - Rounded population estimates 2023 [Available from: <https://www.scotlandscensus.gov.uk/2022-results/scotlands-census-2022-rounded-population-estimates/>.

3.	Minister for Social Care and Mental Wellbeing, Care CSfHaS. Brain Health Service pilot: evaluation - final report: Scottish Government; 2025 [Available from: <https://www.gov.scot/publications/evaluation-pilot-brain-health-service-final-report/pages/3/>.

4.	Brain Health Scotland, Stefano Sd, Borthwick A, Pennington C. Attitudes Towards Brain Health, Dementia Risk Reduction and Early Detection: Brain Halth Scotland 2023 Survey Report. 2024.

5.	Scottish Government. Dementia in Scotland: Everyone's Story. In: Mental Health Directorate, editor.: *Scottish Government,*; 2023.

6.	Rachel Whitmer Lab. STAR - Study of Healthy Aging in African Americans n.d. [cited 2025 2nd of March]. Available from: <https://rachelwhitmer.ucdavis.edu/star>.

7.	Kelly S, Lafortune L, Hart N, et al. Dementia priority setting partnership with the James Lind Alliance: using patient and public involvement and the evidence base to inform the research agenda. *Age Ageing.* 2015;**44**(6):985-93.

8.	Law E, Starr JM, Connelly PJ. Dementia research--what do different public groups want? A survey by the Scottish Dementia Clinical Research Network. *Dementia (London).* 2013;**12**(1):23-8.

9.	Shah H, Albanese E, Duggan C, et al. Research priorities to reduce the global burden of dementia by 2025. *Lancet Neurol.* 2016;**15**(12):1285-94.

10.	Tong A, Synnot A, Crowe S, et al. Reporting guideline for priority setting of health research (REPRISE). *BMC Med Res Methodol.* 2019;**19**(1):243.

11.	Stirland L. Exploring the Priorities of People with Dementia and Multiple Comorbidities: Focus groups to set research priorities for dementia in the context of other conditions: Global Brain Health Institute,; n.d. [cited 2025 3rd of April]. Available from: <https://www.gbhi.org/projects/exploring-priorities-people-dementia-and-multiple-conditions>.

12.	Coleman S, Wray F, Hudson K, et al. Using consensus methods to prioritize modifiable risk factors for development of manifestations of frailty in hospitalized older adults. *Nursing Open.* 2023;**10**(2):1016-28.

13.	Logan M, Leitch S, Bosakh Z, Beishon L, Quinn TJ. Comparing international dementia research priorities-Systematic review. *Int J Geriatr Psychiatry.* 2022;**37**(12).

14.	Eysenbach G. Improving the Quality of Web Surveys: The Checklist for Reporting Results of Internet E-Surveys (CHERRIES). *J Med Internet Res.* 2004;**6**(3):e34.
